# High GTSE1 expression promotes cell proliferation, metastasis and cisplatin resistance in ccRCC and is associated with immune infiltrates and poor prognosis

**DOI:** 10.3389/fgene.2023.996362

**Published:** 2023-03-14

**Authors:** Pu Lei, Mengzhao Zhang, Yan Li, Ziming Wang

**Affiliations:** ^1^ Department of Urology, The Second Affiliated Hospital of Xi’an Jiaotong University, Xi’an, Shanxi, China; ^2^ Department of Urology, Yulin City No. 2 Hospital, Yulin, Shaanxi, China; ^3^ Department of Vascular Surgery, The First Affiliated Hospital of Xi’an Jiaotong University, Xi’an, Shaanxi, China

**Keywords:** GTSE1, ccRCC, prognostic biomarkers, immune infiltration, malignant progression

## Abstract

**Background:** Clear cell renal cell carcinoma is the most common and fatal form of kidney cancer, accounting for 80% of new cases. Although it has been reported that GTSE1 is highly expressed in a variety of tumors and associated with malignant progression and poor clinical prognosis, its clinical significance, correlations with immune cell infiltration and biological function in ccRCC are still poorly understood.

**Methods:** The gene expression, clinicopathological features, and clinical significance of GTSE1 were analyzed using multiple databases, including TCGA, GEO, TIMER, and UALCAN Kaplan–Meier survival analysis, gene set enrichment analysis gene ontology enrichment Gene Ontology, and Kyoto Encyclopedia of Genes and Genomes (KEGG) were performed. Tumor-infiltrating immune cells and immunomodulators were extracted and analyzed using TCGA-KIRC profiles. Protein‒protein interactions were built using the STRING website. The protein level of GTSE1 in ccRCC patients was detected by immunohistochemistry using a ccRCC tissue chip. Finally, MTT assays, colony-formation assays, cell flow cytometry analyses, EdU-staining assays, wound-healing assays, and transwell migration and invasion assays were conducted to assess the biological function of GTSE1 *in vitro*.

**Results:** GTSE1 was overexpressed in ccRCC tissues and cells, and GTSE1 overexpression was associated with adverse clinical-pathological factors and poor clinical prognosis. Meanwhile, the functional enrichment analysis indicated that GTSE1 and its coexpressed genes were mainly related to the cell cycle, DNA replication, and immunoreaction, such as T-cell activation and innate immune response, through multiple signaling pathways, including the P53 signaling pathway and T-cell receptor signaling pathway. Furthermore, we observed a significant relationship between GTSE1 expression and the levels of infiltrating immune cells in ccRCC. Biological functional studies demonstrated that GTSE1 could promote the malignant progression of ccRCC by promoting cell proliferation, cell cycle transition, migration, and invasion capacity and decreasing the sensitivity of ccRCC cells to cisplatin.

**Conclusion:** Our results indicate that GTSE1, serving as a potential oncogene, can promote malignant progression and cisplatin resistance in ccRCC. Additionally, high GTSE1 expression contributes to an increased level of immune cell infiltration and is associated with a worse prognosis, providing a potential target for tumor therapy in ccRCC.

## Introduction

Renal cell carcinoma (RCC) is the most common type of kidney cancer, accounting for approximately 90% of cases, and one of the most common malignant tumors of the adult urinary system, accounting for approximately 2.2% of cases (Barata and Rini, 2017; Sung et al., 2021). The incidence of renal cell carcinoma (RCC) is gradually increasing, and the mortality rate has become the highest among all urological cancers (Capitanio et al., 2019). Clear cell renal cell carcinoma (ccRCC) is the most common histological type, accounting for approximately 85% of RCCs (Padala and Kallam, 2022). In view of the insensitivity of renal clear cell carcinoma to radiotherapy and chemotherapy, surgical operation is still the dominant treatment method, although the postoperative recurrence rate is still nearly 40%. In recent years, immunotherapy-based combinations have become the standard of treatment for patients with metastatic RCC and have shown effectiveness and improved overall survival in the first-line metastatic scenario (Lv et al., 2021). As a heterogeneous disease, ccRCC still lacks effective biomarkers for individualized treatment methods, especially in current immunotherapy. As a result, finding new immune-related molecular biomarkers and treatment targets for ccRCC is critical to improve the prognosis of ccRCC and obtain intervention benefits in patients.

The connection and interaction between the tumor microenvironment (TME) and tumor cells play a vital role in tumor occurrence, development, and recurrence. The TME, consisting of tumor cells and a variety of stromal cells, immune cells, cytokines, and chemokines, is essential for the *in situ* proliferation, directed metastasis, and immune microenvironment modification of tumor cells (Hinshaw and Shevde, 2019). ccRCC is more prone to infiltration of immune cells and alteration of the immune microenvironment, which is more conducive to the malignant progression of tumors. Therefore, immunotherapy in ccRCC also shows its potential application value. Immunotherapy has become an important complementary therapy in addition to radiotherapy, chemotherapy, and surgical treatment, mainly for patients with advanced or metastatic ccRCC (Dong et al., 2020). Immunotherapy represented by immune checkpoint (PD-1, CTLA4) inhibitors has made promising progress in the treatment of ccRCC and can prolong the overall survival (OS) and improve patient prognosis (Atkins and Tannir, 2018). However, up to 70% of patients with ccRCC still do not respond to immunosuppressive agents; thus, finding more reliable and effective immune-related biomarkers and prognostic markers is extremely urgent and necessary.

GTSE1, also known as G2 and S phase expressed-1, is mainly located in the cytoplasm and is specifically expressed in the G2 and S phases of the cell cycle (Monte et al., 2000). GTSE1 has a tight relationship with microtubules and can suppress P53-induced apoptosis and promote the malignant proliferation of tumors by promoting the degradation of P53 (Lin et al., 2019). Current studies have confirmed that GTSE1 expression is upregulated in a variety of tumors and is also associated with worse prognosis in tumor patients. For instance, GTSE1, as a cell cycle-associated protein, exerts a proliferative role in multiple tumors, such as prostate cancer, lung cancer, and bladder cancer (Liu et al., 2019; Zhang F. et al., 2021; Lai et al., 2021). GTSE1, also known as a regulated cytoskeletal protein, can promote cell migration and invasion in cervical cancer and hepatocellular carcinoma (HCC) (Wu et al., 2017; Chen et al., 2021). Considering its close relationship with microtubules, GTSE1 can promote the progression of osteosarcoma by inducing DNA repair and cisplatin resistance (Xie et al., 2021). Meanwhile, GTSE1 can also promote the malignant biological behavior of hepatocellular carcinoma by reducing the sensitivity of HCC cells to 5-FU (Wu et al., 2017). Many studies have confirmed the carcinogenic effect of GTSE1 in tumors. However, to date, the biological role and underlying molecular mechanism of GTSE1 in ccRCC are still poorly understood.

In this research, we first investigated the expression profiles of GTSE1 in ccRCC and identified its biological functions, potential clinical value, and relationship with tumor-infiltrating immune cells (TIICs) in ccRCC. In summary, we demonstrated that GTSE1 was abnormally highly expressed in ccRCC tissues based on the IHC assay of a ccRCC tissue chip and multiple databases analyses, including TCGA and GEO. Meanwhile, GTSE1 overexpression was associated with adverse clinical-pathological factors, worse outcomes, and malignant phenotypes. However, the TME-related and functional enrichment analyses verified that there was a positive relationship between GTSE1 expression and immune cell infiltration in the ccRCC microenvironment. A series of functional experiments also confirmed that high GTSE1 expression could promote the malignant biological behavior of ccRCC *in vitro*. Conclusively, our study indicated that GTSE1 may serve as a prognostic biomarker and a novel immune-associated therapeutic target for ccRCC patients.

## Materials and methods

### Data acquisition and processing

Five mRNA microarray datasets (GSE68417, GSE76351, GSE16449, GSE46699, GSE40435) were obtained from the NCBI-GEO datasets (https://www.ncbi.nlm.nih.gov/geo/). TCGA-KIRC datasets (611 cases, N = 72, T = 539) containing the gene expression level and its corresponding clinical information were derived from the TCGA database (https://portal.gdc.cancer.gov/). For GEO and TCGA cohort, the gene expression was normalized as the log_2_ (TPM+1). The TCGA and GEO publishing criteria were strictly followed in this research.

### Differential expression analysis of GTSE1

GTSE1 expression in the pan-cancer was analyzed *via* the TIMER database and UALCAN web tool based on the TCGA cohort (Li et al., 2017). TCGA paired and unpaired analyses of GTSE1 were visualized in diagrams to exhibit the differential expression between the ccRCC tissues and normal tissues based on the TCGA-KIRC database. GEO datasets (GSE68417, GSE76351, GSE16449, GSE46699, GSE40435) were also utilized for visualization of the differential expression of GTSE1 between the ccRCC tissues and normal tissues.

### Clinicopathological features and survival analysis

The clinicopathologic features including patient’s age, gender, cancer subtypes, metastasis status, cancer stages, and grade were visualized in Box-Whisker plots using the UALCAN website based on the TCGA-KIRC cohort, and the difference between the two groups is established by Student’s t-test and the *p*-value <0.05 is considered as a statistically significant threshold. The clinical outcome of overall survival (OS), Disease-Specific Survival (DSS), and Progress Free Interval (PFI) were selected from the TCGA-KIRC clinical data and The Kaplan-Meier (K-M) curves were utilized to create the survival plots based on the “survival” package and the log-rank test was used to compare the difference in survival curves (cutoff by the median expression level of GTSE1).

### Protein-protein interaction comprehensive analysis

The protein and protein interactions were analyzed using the online website “STRING” (https://cn.string-db.org/). We can get the interacting proteins based on the functional and physical protein associations or the proteins which were part of a physical complex. GTSE1 was imported in the STRING and the protein and protein interaction network was exported based on the confidence score. The proteins whose confidence score ≥0.9 were identified as having the highest confidence were extracted and listed beside the picture.

### Co-expression analysis in LinkedOmics

Linkedomics (http://linkedomics.org/) is an online analysis platform based on the TCGA database which could analyze 32 types of cancer data online (Vasaikar et al., 2018). We selected TCGA-KIRC, RNA-seq, GTSE1, RNA-seq, and Spearman to analyze positive and negative gene sets related to GTSE1 expression, and we set *p*-value <0.05 as the threshold of statistical difference. We took the expression of GTSE1 as the standard to display the positively correlated genes and negatively correlated genes in the form of a volcano map. Meanwhile, the top 50 positively correlated and negatively correlated genes were displayed in the heat map respectively. Finally, GEPIA2 (http://gepia2.cancer-pku.cn) was used to analyze the correlation between the expression level of the top 50 differential genes and the clinical prognosis in ccRCC, and the results were presented in heat map form (Tang et al., 2019). *p < 0.05 indicated statistical significance.

### Functional enrichment analysis

In order to investigate the underlying biological functions of GTSE1. The top 1,000 co-expressed genes obtained from the LinkedOmics were extracted and performed the GO annotation and Kyoto Encyclopedia of Genes and Genomes (KEGG) pathway enrichment analyses. The “clusterProfiler” package was used to perform the functional enrichment analysis including the Gene Ontology (GO) and KEGG analysis. GO annotation consisted of biological process (BP), cellular component (CC), and Molecular function (MF). *p*-value < 0.05 and false discovery rate (FDR) < 0.05 were considered to be statistically significant. The “ggplot2” package was used for visualization.

### Gene set enrichment analysis

The 539 ccRCC patients, whose gene expression data were obtained from the TCGA database were divided into the GTSE1 high-expression group and GTSE1 low-expression group according to the median expression level of GTSE1 with each group containing 269 patients. The gene expression of the two groups was imported to the GSEA 4.2.0 software to analyze the significantly changed signaling pathways and get the top 100 co-expressed genes of GTSE1in each group. The hallmark of gene sets and KEGG pathways were selected for further analysis (https://www.gsea-msigdb.org/gsea/msigdb/collections.jsp#H). Meanwhile, the Nominal *p*-value < 0.05 and FDR q value < 0.25 were set as a threshold of statistically significant.

### TME related analysis

Estimate is an algorithm that predicts tumor purity by calculating the immune score and stroma score based on TCGA expression profile and single-sample Gene Set Enrichment Analysis (ssGSEA) analysis (Yoshihara et al., 2013). The abundance of 28 cell types was estimated and tumor purity, immune score, stromal score, and estimate score were also calculated based on the expression of GTSE1 (cutoff by the median expression level of GTSE1). CIBERSORT is an R/web version tool for deconvolution of expression matrices of human immune cell subtypes based on Linear Support Vector regression. The approach is based on a known reference set that provides a gene expression signature set for 22 immune cell subtypes: LM22 (Newman et al., 2015). The degree of infiltration of 22 immune cells was also grouped and calculated according to the median expression of GTSE1. The correlation between the HRD (homologous recombination deficiency) and GTSE1 expression was analyzed by the means of the “fmsb” package and Spearman’s method. Finally, the correlation between the immune checkpoint and GTSE1 expression was calculated and displayed as a heat map.

### Cell culture and transfection

The ccRCC cell lines (786-O, Caki-1, RCC-4, SW839, 769-P, and OS-RC-2) and Human renal tubular epithelial cell line (HK-2) were all purchased from the American Type Culture Collection (ATCC, Manassas, VA, USA). All the cells mentioned above were cultured in RPMI-1640 contained with 10% fetal bovine serum (FBS) and 1% penicillin-streptomycin solution (Gibco, Grand Island, NY, USA) at 37°C in a 5% CO_2_ cell incubator. All cell lines used in the research were at early passages. To suppress the expression of GTSE1, the two double-stranded siRNA oligonucleotides against GTSE1 were designed and chemically synthesized (Shanghai GenePharma Co.) and the siRNA was transfected into the cells using the Lipofectamine 2000 reagent (Thermo Fisher Scientific, Inc). The selected targeting sequences were as follows: si-NC: 5′-UUC​UCC​GAA​CGU​GUC​ACG​UTT-3’; si-GTSE1: 5′-GGA​AUC​AUG​CAC​UGC​UCA​UTT-3’. To upregulate the GTSE1 expression, a GTSE1 overexpression plasmid based on a pcDNA 3.1 vector was designed and synthesized (Beijing, Sino Biological). According to the manufacturer’s protocol, the plasmid GTSE1-overexpression and the negative control plasmid were transfected into the cells using the X-treme GENE HP DNA Transfection Reagent (Roche, Switzerland).

### ccRCC tissue chip and immunohistochemistry (IHC) assays

To investigate the abnormal expression of GTSE1 in ccRCC tissues and the normal adjacent tissues, the ccRCC tissue chip (Catalog No. HKid-CRCC060PG-01) was purchased from Outdo Biotech Co., Ltd (Shanghai, China) including 30 ccRCC tissues and corresponding adjacent non-cancerous tissues. The tissue chip was subjected to the immunohistochemistry staining assay according to the protocol previously described in this research (Zhang M. et al., 2021). Especially, the primary antibody was purchased from Abcam (anti-GTSE1,1:500) (ab272670). Finally, the IHC staining images were scored based on the staining intensity (0, 1, 2+, 3+) and the percentage of positive cells (0 (0%), 1 (1%–25%), 2 (26%–50%), 3 (51%–75%) and 4 (76%–100%)). The final score used to assess the expression level of GTSE1 was calculated by the combination of the two scores, negative (0 score), weak (one to four score), moderate (five to eight score), and strong (9–12 score).

### Quantitative RT-PCR (qRT-PCR)

The total RNA was extracted and isolated from the cell lysis using the RNAfast 200 reagents (Feijie Biotechnology, Shanghai, China) and reversed transcribed into cDNA using the Prime Script RT-PCR kit (Takara Bio Dalian, China). SYBR qPCR Master Mix was used to amplify the cDNA using the CFX96 Real-Time PCR system (Bio-Rad, CA, USA). All the specific primers used in the research were listed as GTSE1, F: CCA​CCG​GGA​TGT​TCT​CCC​T. R: TTC​AGC​CCC​AAC​TTG​TTT​GGA. GAPDH, F: ACC​CAG​AAG​ACT​GTG​GAT​GG. R: CAG​TGA​GCT​TCC​CGT​TCA​G. GAPDH was used as a loading control.

### Western blot assay

The total protein was extracted with RIPA lysis (Catalog Number P0013B, Beyotime, China) containing 0.1 M PMSF and 1% protease inhibitor and phosphatase inhibitor (Shanghai Epizyme Biomedical Technology Co., Ltd.). After denatured by boiling for 10min and mixing with 5x loading buffer, the proteins were separated with SDS-PAGE and transferred onto the 0.45 μm polyvinylidene fluoride (PVDF) membranes. The membranes were subjected to the primary antibodies of anti-GTSE1 (ABclonal, Cat NO A13903, 1:1000) and GAPDH (ABclonal, Cat NO AC001, 1:10,000) at 4°C overnight after being blocked with the 5% nonfat milk for 1 h. On the second day, after being washed with TBST three times, the membranes were incubated with the corresponding peroxidase-conjugated secondary antibody for 1 h at room temperature. Finally, the expression of indicated proteins was detected and visualized by the ECL chemiluminescent detection system (Bio-Rad, CA, USA).

### MTT assay

MTT assay was conducted to evaluate the cell viability of the ccRCC cells under the indicated conditions. Cells in the logarithmic growth phase were digested and centrifuged and then planted at a density of 4,000 cells per well into a 96-well plate with each well containing 200 µL culture medium. After cultivating for a certain time, the supernatant was removed and 200 µL complete culture medium containing 0.5 mg/mL MTT was added into each well. The 96-well plate was placed in a cell incubator for further cultivation for 4 h and then the OD value at 450 nm was detected with an ELISA reader (Bio-Rad, Hercules, CA, USA) after the 96 well-plates were shaken for 10 min with each well containing 150 µL DMSO. The experiment was executed in triplicate.

### Clone formation assay

Cells were digested and centrifuged and then seeded in six well-plates with 1,000 cells in each well. Six well plates were cultured in the cell incubator for approximately 10 days to make the clones visual. After washing with ice-cold PBS three times, fixed and stained with 0.1% crystal dissolved with 4% paraformaldehyde for 10 min, the cell colony was captured with a microscope (Olympus, Tokyo, Japan). The experiment was repeated three times.

### Cell flow cytometry analysis

For apoptosis analysis, cells planted in six well-plates were washed with PBS and harvested in ice-cold PBS. After being washed with PBS three times, the cells were suspended in a binding buffer containing Annexin V and PI staining solution at dark for 15min. Finally, the percentage of apoptosis cells was detected by flow cytometry (BD, Biosciences, USA) according to the manufacturer’s protocols. For cell cycle distribution analysis, cells in six well-plates were digested with trypsin, washed with ice-cold PBS, fixed with 70% ethanol at 4 °C for 12 h, and then stained with propidium iodide (PI, 50 μg/mL) and RNase (100 μg/mL) in PBS at dark for 15min. The cell cycle distribution was detected and analyzed by flow cytometer (BD, Biosciences, USA) and cell quest software version 3.3 (BD, Biosciences) according to the manufacturer’s protocols. The experiments were conducted three times.

### Wound healing assay

Cells were seeded into six well-plates and then scratched a distance with a 200 μL pipette when the cell density reached 100%. After changing to serum-free medium, six well-plates were placed in a cell incubator for various durations (24 h and 48 h). The images of the scratch were captured by an orthotopic microscope every 24 h until the distance almost disappeared. This experiment was repeated in triplicate.

### Transwell migration and invasion assay

Boyden chambers (Millipore, Germany) with an 8-μm pore size were placed into 24 well-plates to assess the migration and invasion ability. Briefly, 4 × 10^4^ cells seeded in the upper chamber suspended in 200 μL serum-free culture medium were used to evaluate the migration ability of ccRCC cells. Meanwhile, 8 × 10^6^ cells seeded in the upper chamber with Matrigel suspended in 200 μL serum-free culture medium was used to assess the invasion ability. After being incubated in the cell incubator for a certain time, the chambers were washed with PBS, fixed, and stained with 0.1% crystal violet dissolved with 4% paraformaldehyde. The visible cells were captured and counted with an inverted light microscope at ×100 magnification in five random fields. The experiments were performed in triplicate.

### Statistical analysis

All statistical analyses and visualization of the results shown in this research were executed by R software version 4.1.3 and Prism version 9.0. The Wilcoxon test was used to compare two groups, whereas the Kruskal–Wallis test was used to compare multiple groups. Overall survival, Disease-Specific Survival, and Progress Free Interval were performed using the Kaplan–Meier curves and the log-rank test. Spearman analysis was used to evaluate the correlation coefficient among variances in this research. The statistical difference between the two groups was analyzed with Student’s t-test. *p-value < 0.05 was considered as the threshold of statistically significant.

## Results

### High expression of GTSE1 in ccRCC

**FIGURE 1:**
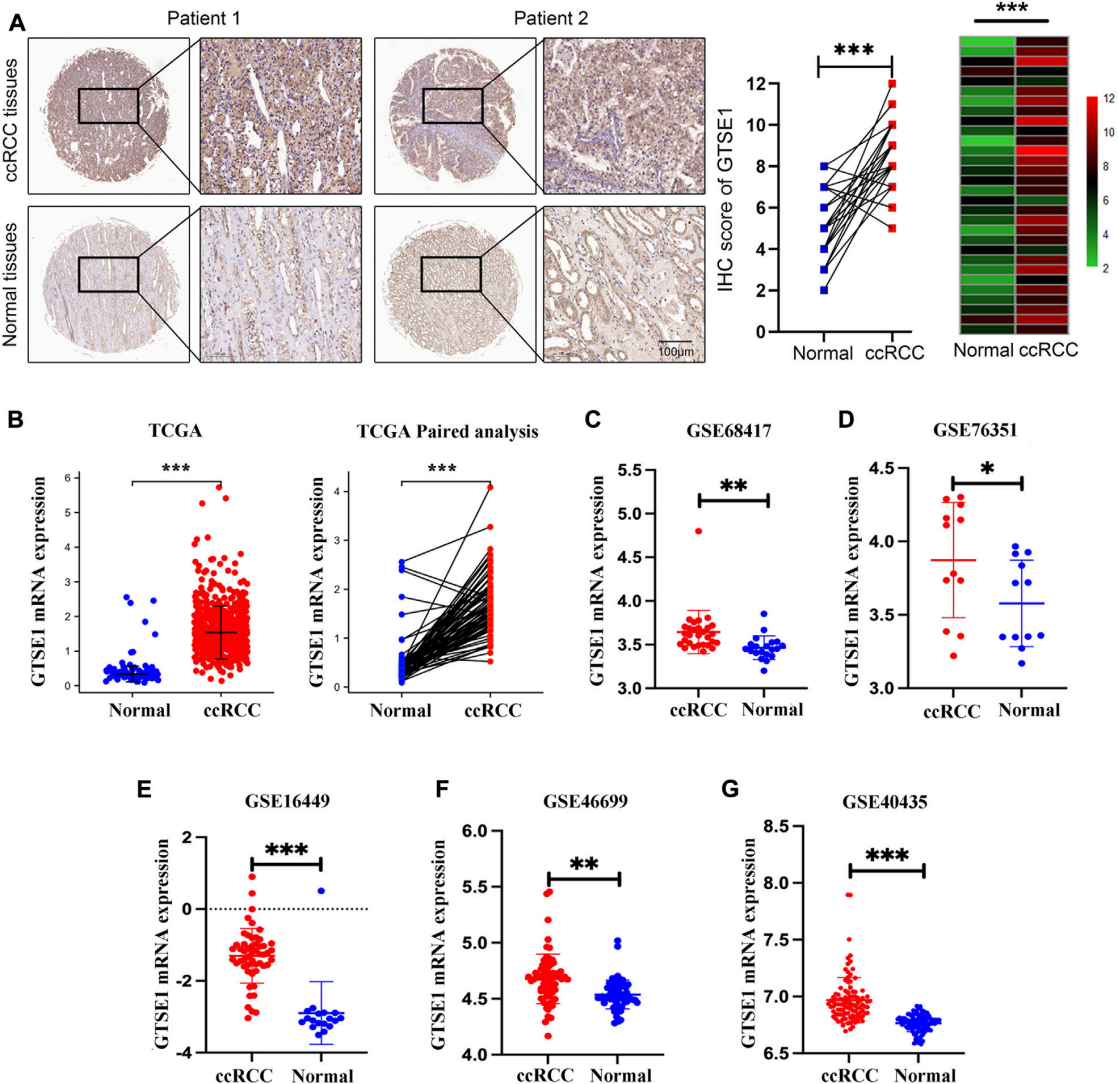
High expression of GTSE1 in ccRCC. **(A)** Representative images of IHC staining of GTSE1 in ccRCC tissues and matched normal tissues (n = 30). The IHC score of each tissue in ccRCC tissues and matched normal tissue were analyzed and exhibited in scatter diagrams and heatmaps. **(B)** GTSE1 mRNA level in ccRCC samples was shown in TCGA paired and unpaired analysis. **(C–G)** GTSE1 mRNA levels in ccRCC tissues and normal tissues in GSE68417 **(C)**, GSE76351 **(D)**, GSE16449 **(E)**, GSE46699 **(F)** and GSE40435 **(G)**. *p < 0.05, **p < 0.01, ***p < 0.001.

GTSE1 mRNA expression was investigated across cancers using the TIMER 2.0 web tool and the UALCAN web tool. The data indicated that GTSE1 expression was upregulated in a variety of tumor tissues, such as kidney renal clear cell carcinoma (ccRCC), bladder cancer (BLCA), breast cancer (BRCA), cholangiocarcinoma (CHOL), colon adenocarcinoma (COAD), esophageal carcinoma (ESCA), and head and neck squamous cell carcinoma (HNSC) (Supplementary Figure S1). To further explore GTSE1 expression in ccRCC, a ccRCC tissue chip consisting of 30 tumor tissues and matched normal tissues from 30 ccRCC patients was subjected to immunohistochemical staining, which showed that GTSE1 was overexpressed in ccRCC tissues compared with the corresponding normal tissues (Figure 1A). Meanwhile, the TCGA database and GEO database were also used to clarify the differential expression of GTSE1 in ccRCC compared with normal kidney tissues. We found that GTSE1 was upregulated in ccRCC samples in TCGA paired and unpaired analysis when compared with normal samples (Figure 1B). The GEO database also showed that GTSE1 expression in ccRCC tissues was higher than that in normal tissues (Figures 1C–G). Overall, all these above results demonstrated that GTSE1 was overexpressed in ccRCC tissues compared with normal tissues.

### GTSE1 expression in subgroups of different clinical characteristics

**FIGURE 2:**
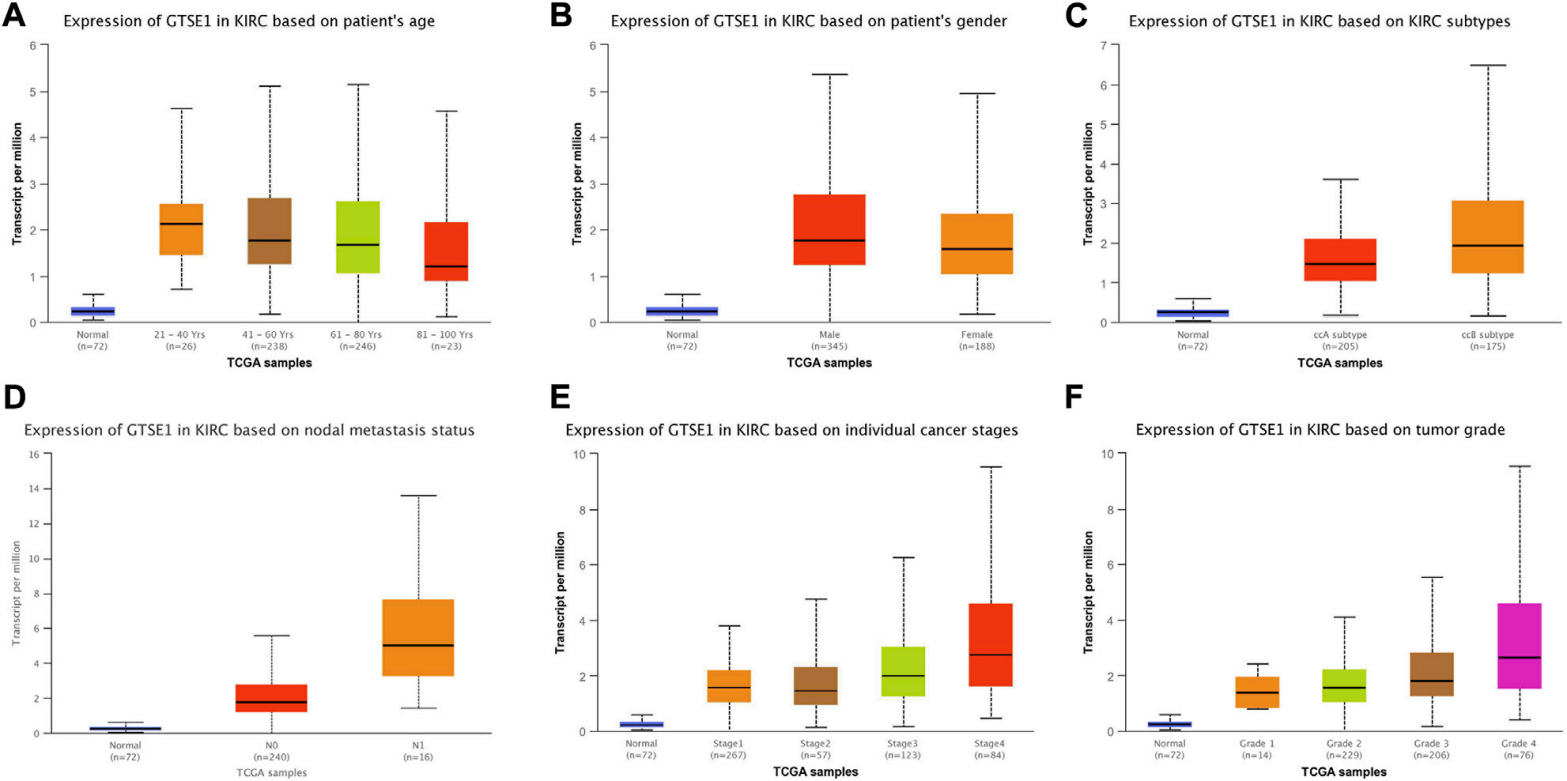
GTSE1 expression in subgroups of different clinical characteristics. Box plots showing the relative mRNA expression of GTSE1 in different groups of ccRCC patients: **(A)** patient age **(B)**, patient gender **(C)** ccRCC subtypes, **(D)** nodal metastasis status, **(E)** individual cancer stages, and **(F)** tumor grade.

The UALCAN web tool based on the TCGA-KIRC database was used to analyze the association between GTSE1 expression and clinicopathological parameters in ccRCC. The data suggested that GTSE1 expression was significantly correlated with KIRC subtype, cancer stage, nodal metastasis, and tumor grade (Figures 2C–F). Briefly, higher GTSE1 expression correlated with more lymph node metastasis, advanced clinical stages, and higher tumor grades. According to the gene microarray data and different clinical prognoses, two distinct subtypes (ccA and ccB) were used to distinguish ccRCC. Generally, tumor patients with the ccA subtype usually have a better prognosis than those with the ccB subtype (Brannon et al., 2010). Concordantly, there was a higher expression of GTSE1 in the ccB subtype than in the ccA subtype. Non-etheless, significant differences were not observed among GTSE1 expression and clinical-pathological features, such as patient age and gender (Figures 2A,B). The correlation between GTSE1 expression and the clinicopathological parameters of ccRCC is summarized in Table 1. Similar results were also observed in Table 2 based on the TCGA-KIRC cohort analysis. There was a positive correlation between GTSE1 expression and tumor stage, lymph node metastasis, distant organ metastasis, pathologic stage, and histologic grade. These results suggested that GTSE1 expression was positively associated with adverse clinical-pathological parameters, and the higher expression of GTSE1 indicated an advanced malignant progression of ccRCC.

**TABLE 1:** The correlation between GTSE1 expression and different clinical characteristics.

Variables	Subgroup	No.	Comparisons	Statistical significance (*P*)
Sample	Normal	72	Normal vs. primary tumor	1.62E-12
	Primary tumor	533		
Patient's age	Normal	72		
	21-40 years	26	Normal-vs-Age(21-40Yrs)	2.75E-04
	41-60 years	238	Normal-vs-Age(41-60Yrs)	2.34E-14
	61-80 years	246	Normal-vs-Age(61-80Yrs)	1.62E-12
	81-100 years	23	Normal-vs-Age(81-100Yrs)	1.48E-03
Patient's gender	Normal	72		
	Male	345	Normal-vs-Male	1.11E-16
	Female	188	Normal-vs-Female	1.11E-16
KIRC subtypes	Normal	72	Normal-vs-ccA subtype	1.62E-12
	ccA	205	Normal-vs-ccB subtype	1.63E-12
	ccB	175	ccA subtype-vs-ccB subtype	2.39E-06
Nodal metastasis	Normal	72	Normal-vs-N0	9.99E-16
	N0	240	Normal-vs-N1	4.14E-05
	N1	16	N0-vs-N1	6.16E-04
Cancer stage	Normal	72		
	Stage 1	267	Normal-vs-Stage1	1.62E-12
	Stage 2	57	Normal-vs-Stage2	5.59E-07
	Stage 3	123	Normal-vs-Stage3	8.09E-12
	Stage 4	84	Normal-vs-Stage4	9.93E-09
Tumor grade	Normal	72		
	Grade 1	14	Normal-vs-Grade 1	4.19E-04
	Grade 2	229	Normal-vs-Grade 2	<1E-12
	Grade 3	206	Normal-vs-Grade 3	<1E-12
	Grade 4	76	Normal-vs-Grade 4	6.01E-08

**TABLE 2:** Relationship between GTSE1 expression and clinicopathological features in patients with ccRCC.

Characteristic	Low expression of GTSE1	High expression of GTSE1	P	method
Age, n (%)			0.245	Chisq.test
<=60	127 (23.6%)	142 (26.3%)		
>60	142 (26.3%)	128 (23.7%)		
Race, n (%)			0.420	Fisher.test
Asian	3 (0.6%)	5 (0.9%)		
Black or African				
American	33 (6.2%)	24 (4.5%)		
White	231 (43.4%)	236 (44.4%)		
Gender, n (%)			0.116	Chisq.test
Female	102 (18.9%)	84 (15.6%)		
Male	167 (31%)	186 (34.5%)		
T stage, n (%)			**< 0.001**	Chisq.test
T1	155 (28.8%)	123 (22.8%)		
T2	42 (7.8%)	29 (5.4%)		
T3	71 (13.2%)	108 (20%)		
T4	1 (0.2%)	10 (1.9%)		
N stage, n (%)			**0.002**	Chisq.test
N0	121 (47.1%)	120 (46.7%)		
N1	1 (0.4%)	15 (5.8%)		
M stage, n (%)			**< 0.001**	Chisq.test
M0	227 (44.9%)	201 (39.7%)		
M1	23 (4.5%)	55 (10.9%)		
Pathologic stage, n (%)			**< 0.001**	Chisq.test
Stage I	151 (28.2%)	121 (22.6%)		
Stage II	36 (6.7%)	23 (4.3%)		
Stage III	57 (10.6%)	66 (12.3%)		
Stage IV	25 (4.7%)	57 (10.6%)		
Histologic grade, n (%)			**< 0.001**	Chisq.test
G1	9 (1.7%)	5 (0.9%)		
G2	136 (25.6%)	99 (18.6%)		
G3	94 (17.7%)	113 (21.3%)		
G4	24 (4.5%)	51 (9.6%)		

### Prognostic value of GTSE1 in ccRCC

**FIGURE 3:**
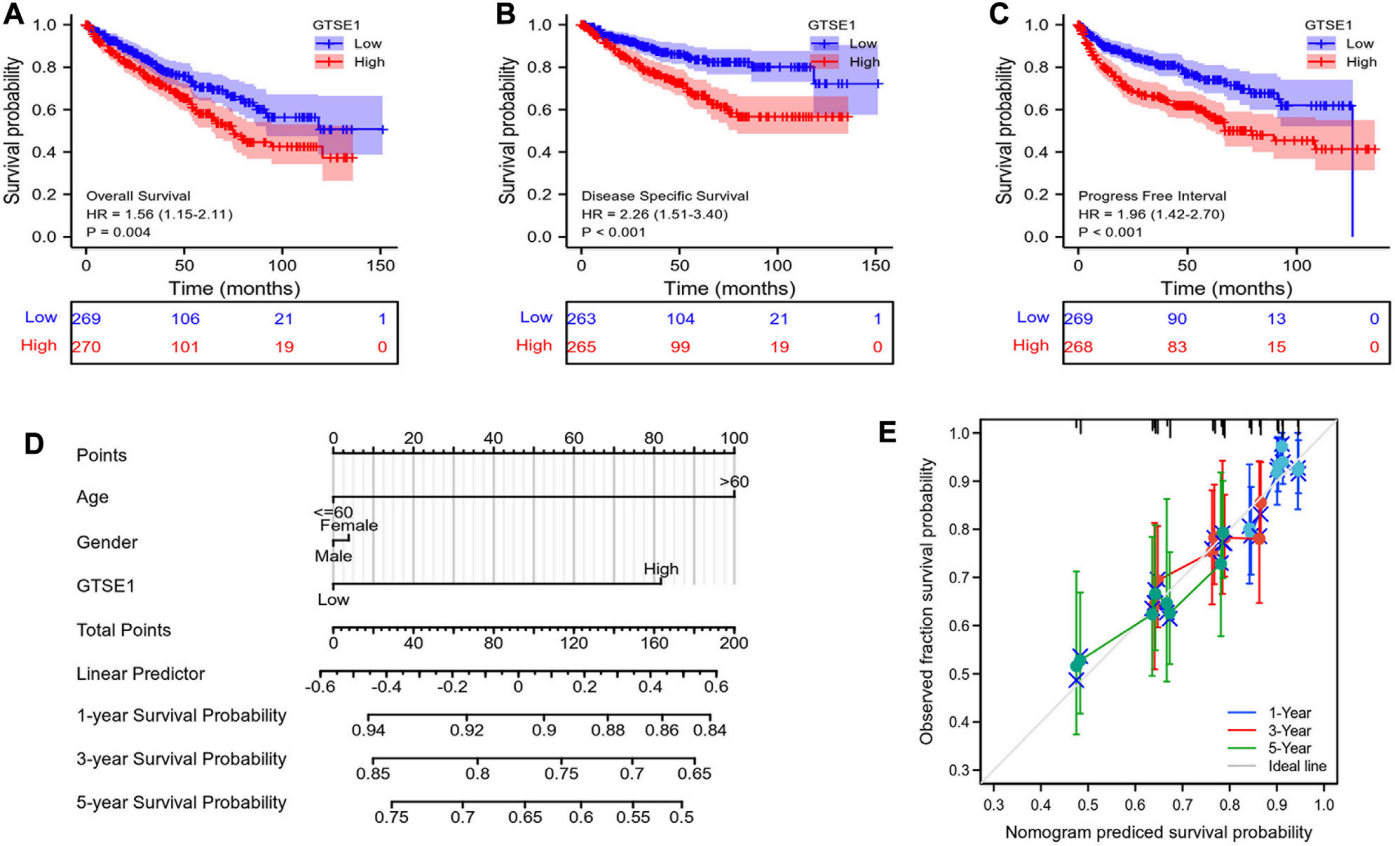
Prognostic value of GTSE1 in ccRCC. Kaplan–Meier survival curve analysis of OS **(A)**, DSS **(B)** and PFI **(C)** verified the prognostic value of GTSE1 in ccRCC. **(D)** Nomogram used to predict the probability of 1-, 3-, and 5-year OS for ccRCC patients based on the patient’s age, gender, and GTSE1 expression level. **(E)** Calibration plot of the nomogram for predicting the OS likelihood.

Then, the relationship between GTSE1 expression and survival outcomes in ccRCC patients was explored by Kaplan–Meier survival curves based on the TCGA-KIRC cohort. The patients were divided into two groups by the median GTSE1 expression. The Kaplan–Meier curves of OS, DSS, and PFI demonstrated that the patients with higher GTSE1 expression had a worse outcome than those with lower GTSE1 expression (OS, HR = 1.56 (1.15–2.11, log-rank *p* = 0.004; DSS, HR = 2.26 (1.51–3.40), log-rank *p* < 0.001; PFI, HR = 1.96 (1.42–2.70), log-rank *p* < 0.001) (Figures 3A–C). The association between GTSE1 expression and clinical prognosis (OS, DSS, PFI) across cancers was also analyzed and is shown in Supplementary Figure S2. To assist clinicians in quickly determining the clinical overall survival of ccRCC patients, we designed a multivariate Cox analysis nomogram based on patient age, gender, and GTSE1 expression (Figure 3D). Briefly, we scored ccRCC patients on a scale from 0 to 100 based on their age, gender, and GTSE1 expression and then calculated the overall score. The total scores were then plotted on the horizontal axis to correspond to the survival probability of ccRCC patients after 1, 3, and 5 years. Meanwhile, calibration was also used to illustrate the accuracy of the nomogram model. The abscissa is the survival probability predicted by the model, and the ordinate is the survival probability actually observed. Each point represents the survival probability predicted by the model and the survival probability observed. The gray diagonal is the ideal case line. The bias-corrected line in the calibration plot was getting closer to the ideal curve (also known as the 45-degree line), which shows a reasonable agreement between observed and anticipated values (Figure 3E). The above results fully demonstrated that GTSE1 might serve as a prognostic biomarker associated with worse outcomes in ccRCC.

### Constructing protein interaction networks

**FIGURE 4:**
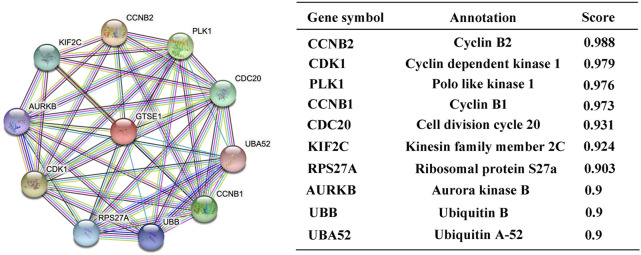
Constructing protein interaction networks. PPI network of GTSE1 and the top 10 proteins interacting with GTSE1. Annotation of GTSE1-interaction proteins and their confidence scores.

Protein‒protein interactions (PPIs) constitute an important part of the cellular biochemical reaction network. Understanding protein-protein interactions is extremely important for understanding the biological functions and molecular mechanisms of proteins. Hence, the STRING web tool was used to analyze the PPI network of GTSE1, and the top 10 proteins interacting with GTSE1 are shown and listed in Figure 4. The proteins sorted according to the combined score were as follows: CCNB2, CDK1, PLK1, CCNB1, CDC20, KIF2C, RPS27A, AURKB, UBB, and UBA52. Previous research has verified that CCNB2, CCNB1, CDK1, PLK1, CDC20, KIF2C, RPS27A, and AURKB play vital roles in regulating the cell cycle transition, radiosensitivity and cell proliferation in various tumors (Wang et al., 2014; Nie et al., 2020; Zou et al., 2020; Gao et al., 2021; Gheghiani et al., 2021; Zhao et al., 2021). From the PPI network of GTSE1, we speculate that GTSE1 plays an important role in the cell cycle transition and malignant proliferation of tumors.

### GTSE1 coexpression networks and functional enrichment analysis

**FIGURE 5:**
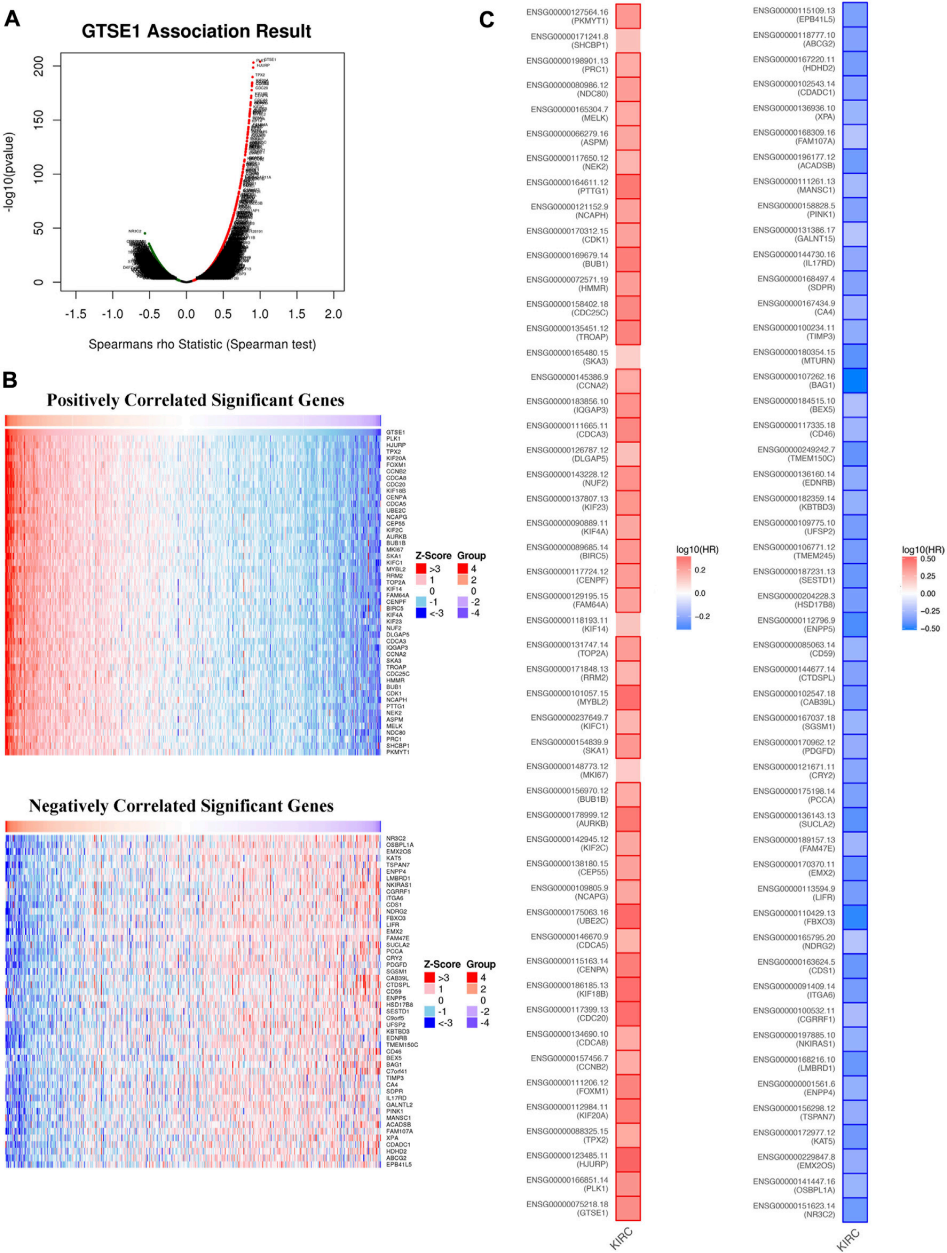
GTSE1 coexpression networks in ccRCC (LinkedOmics). **(A)** The volcano plot of genes highly correlated with GTSE1 based on the Spearman test. **(B)** The top 50 genes positively correlated or negatively correlated with GTSE1 are shown in the heatmaps. **(C)** The overall survival analysis of the top 50 genes positively correlated or negatively correlated with GTSE1 is also displayed in the survival heatmaps.

**FIGURE 6:**
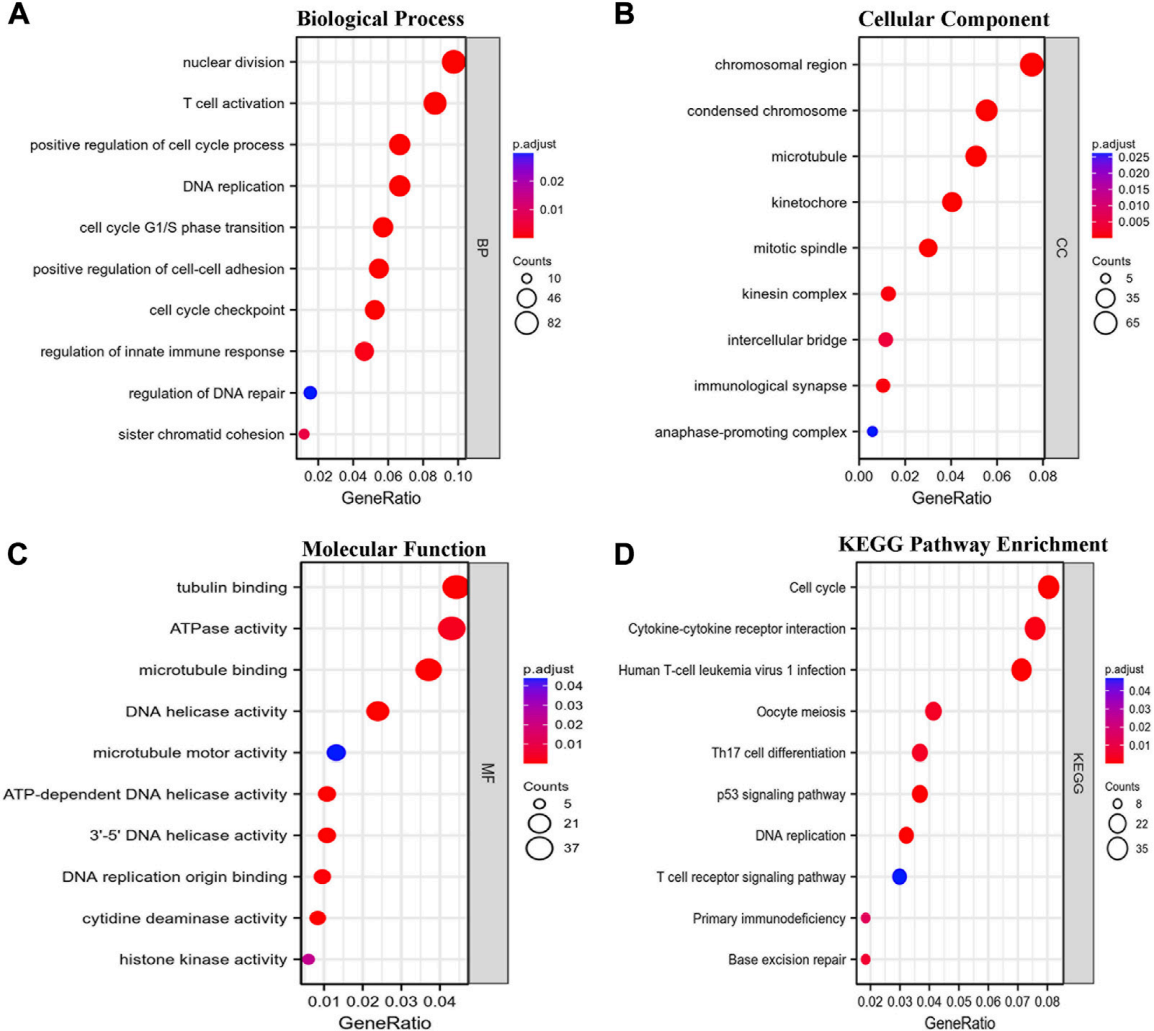
Functional enrichment analysis. GO annotation including biological process (BP) **(A)**, cellular component (CC) **(B)**, and molecular function (MF) **(C)** and Kyoto Encyclopedia of Genes and Genomes (KEGG) **(D)** pathway enrichment analyses based on the coexpressed genes of GTSE1 were clustered, and the top 10 terms of each subtype are displayed in the bubble diagram.

Coexpression gene networks, including positively and negatively regulated genes, can reflect biological functions and underlying signaling pathways. The coexpressed genes of GTSE1 were analyzed using LinkedOmics based on the TCGA-KIRC cohort, and the results are presented in the form of a volcano map (Figure 5A). There were 6,830 genes positively correlated with GTSE1 and 3,560 genes negatively correlated with GTSE1 (FDR <0.01). The top 50 genes positively and negatively correlated with GTSE1 are shown in the heatmaps (Figure 5B). From the heatmaps, we could see a strong positive relationship between GTSE1 and PLK1, HJURP, TPX2, *etc.* Meanwhile, there was also an obvious negative relationship between GTSE1 and NR3C2, OSBPL1A, EMX2OS, *etc.* Remarkably, 46 of the top 50 positively regulated genes might serve as high-risk markers in ccRCC because of their high hazard ratio (HR, *p*-value < 0.05). Meanwhile, all top 50 negatively regulated genes might serve as low-risk markers because of their low HR (*p*-value < 0.05) (Figure 5C). To investigate the biological functions and underlying pathways of GTSE1, GO annotation and KEGG pathway enrichment analyses were performed based on the expression of GTSE1 and its coexpressed genes. Gene ontology consisting of molecular functions (MF), biological process (BP), and cellular component (CC) was clustered and analyzed using the “clusterprofile” package based on “R” software version 4.1.3. (Figures 6A–C). The enrichment analysis of BP indicated that GTSE1 and its coexpressed genes might be involved in the cell cycle transition and immune-related processes, including nuclear division, DNA replication, cell cycle G1/S transition, cell cycle checkpoint, T-cell activation, regulation of innate immune response, *etc.* GTSE1 and its coexpressed genes might be primarily involved in cellular components, including chromosomal regions, condensed chromosomes, microtubes, kinetochores, and immunological synapses. Molecular functions, including tubulin binding, ATPase activity, microtubule binding, DNA helicase activity, DNA replication, origin binding, *etc.*, might have a close relationship with GTSE1 dysregulation. Moreover, the top 10 KEGG pathways that might be regulated by GTSE1 were clustered and are shown in Figure 6D, including the cell cycle, cytokine‒cytokine receptor interaction, oocyte meiosis, P53 signaling pathway, DNA replication, human T-cell leukemia virus one infection, Th17-cell differentiation, T-cell receptor signaling pathway, primary immunodeficiency, and base excision repair. The GO annotation and KEGG analysis revealed that GTSE1 not only participates in the regulation of cell proliferation and cell cycle transition but also has a tight correlation with the immune response.

### GSEA between the high- and low-GTSE1-expression groups

**FIGURE 7:**
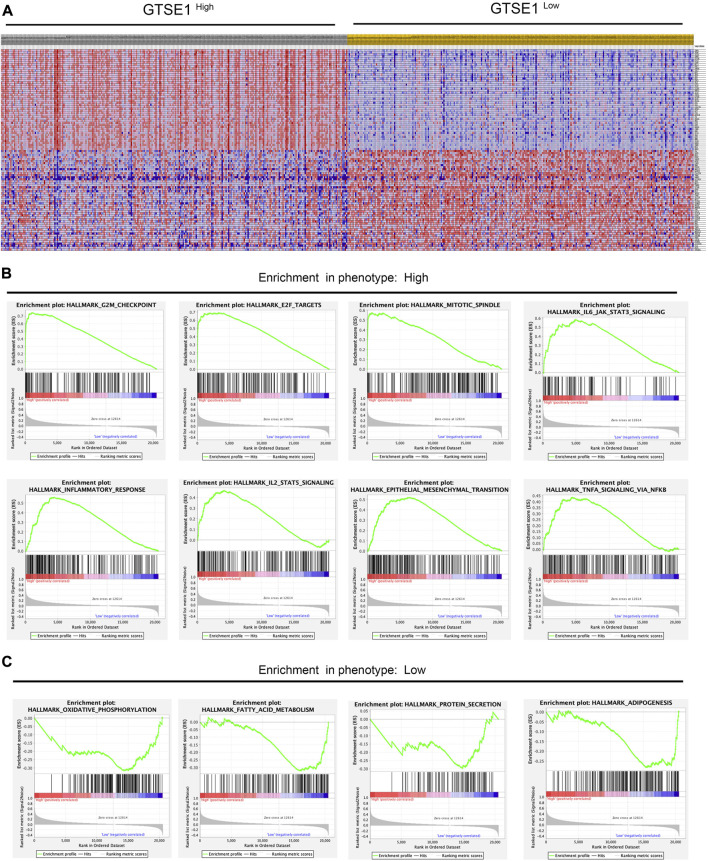
GSEA of GTSE1 in the TCGA-KIRC dataset and the significantly changed pathways in 50 hallmark gene sets based on GTSE1 expression. **(A)** Heatmaps of the top 100 genes upregulated or downregulated in the GTSE1 high-expression group and GTSE1 low-expression group in ccRCC patients. **(B)** GSEA displayed the most significantly upregulated signaling pathways enriched in the GTSE1 high-expression group. **(C)** GSEA displayed the most significantly downregulated signaling pathways enriched in the GTSE1 low-expression group.

According to the median GTSE1 expression in TCGA-KIRC, patients were divided into high- and low-GTSE1-expression groups for GSEA to investigate the potential role and signaling pathways regulated by GTSE1. First, the top 100 genes upregulated in the high-GTSE1-expression group and the top 100 genes downregulated in the low-GTSE1-expression group are presented in the heatmap (Figure 7A). Moreover, 43/50 gene sets were upregulated in the GTSE1-high phenotype, and 7/50 gene sets were upregulated in the GTSE1-low phenotype. The significantly changed phenotype and KEGG pathways enriched in the GTSE1 high-expression group were as follows: “HALLMARK G2M CHECKPOINT”, “HALLMARK F2E TARGETS”, “HALLMARK MITOTIC SPINDLE”, “HALLMARK_IL6 JAK_STAT3_SIGNALING”, “HALLMARK INFLAMMATORY RESPONSE”, “HALLMARK IL2 STAT5 SIGNALING”, “HALLMARK EPITHELIAL MESENCHYMAL TRANSITION”, “HALLMARK_TNFA SIGNALING _VIA _NFKB”, “KEGG CELL CYCLE”, “KEGG_PRIMARY_ IMMUNODEFICIENCY”, “KEGG CYTOKINE RECEPTOR INTERACTION”, “KEGG OOCYTE MEIOSIS”, “KEGG T CELL RECEPTOR SIGNALING PATHWAY”, and “KEGG P53 SIGNALING PATHWAY” (Figure 7B; Supplementary Figure S3) Meanwhile, the phenotype and KEGG pathways enriched in the GTSE1 low-expression group were as follows: “HALLMARK OXIDATIVE PHOSPHORYLATION”, HALLMARK FATTY ACID METABOLISM”, “HALLMARK_PROTEIN_SECRETION”, and “HALLMARK_ ADIPOGENESIS” (Figure 7C) (Table 3). These results suggested that GTSE1 might be involved in epithelial-mesenchymal transition in addition to cell cycle regulation and the immune response. In summary, GSEA further demonstrated that GTSE1 might play a vital role in carcinogenesis and immunomodulation in ccRCC.

**TABLE 3:** The enrichment of GSEA gene sets at both the NOM P-value <0.05 and FDR q-value <0.25.

Enrichment in phenotype: High	ES	NES	NOM P-value	FDR q-value
HALLMARK_G2M_CHECKPOINT	0.74	2.20	0.00	0.00
HALLMARK_E2F_TARGETS	0.69	2.05	0.00	0.00
HALLMARK_MITOTIC_SPINDLE	0.57	1.69	0.00	0.00
HALLMARK_IL6_JAK_STAT3_SIGNALING	0.58	1.65	0.00	0.00
HALLMARK_INFLAMMATORY_RESPONSE	0.56	1.64	0.00	0.00
HALLMARK_TNFA_SIGNALING_VIA_NFKB	0.44	1.29	0.01	0.12
HALLMARK_IL2_STAT5_SIGNALING	0.47	1.38	0.00	0.05
HALLMARK_EPITHELIAL_MESENCHYMAL_TRANSITION	0.52	1.54	0.00	0.01
Enrichment in phenotype: Low	ES	NES	NOM P-value	FDR q-value
HALLMARK_OXIDATIVE_PHOSPHORYLATION	-0.31	-1.48	0.00	0.02
HALLMARK_FATTY_ACID_METABOLISM	-0.32	-1.68	0.00	0.01
HALLMARK_PROTEIN_SECRETION	-0.29	-1.34	0.00	0.05
HALLMARK_ADIPOGENESIS	-0.28	-1.22	0.00	0.12

ES, enrichment score; FDR, false discovery rate; ES, Enrichment Score; NES, normalized enrichment score; NOM, normalized

### Association between GTSE1 expression and immune infiltration in ccRCC

**FIGURE 8:**
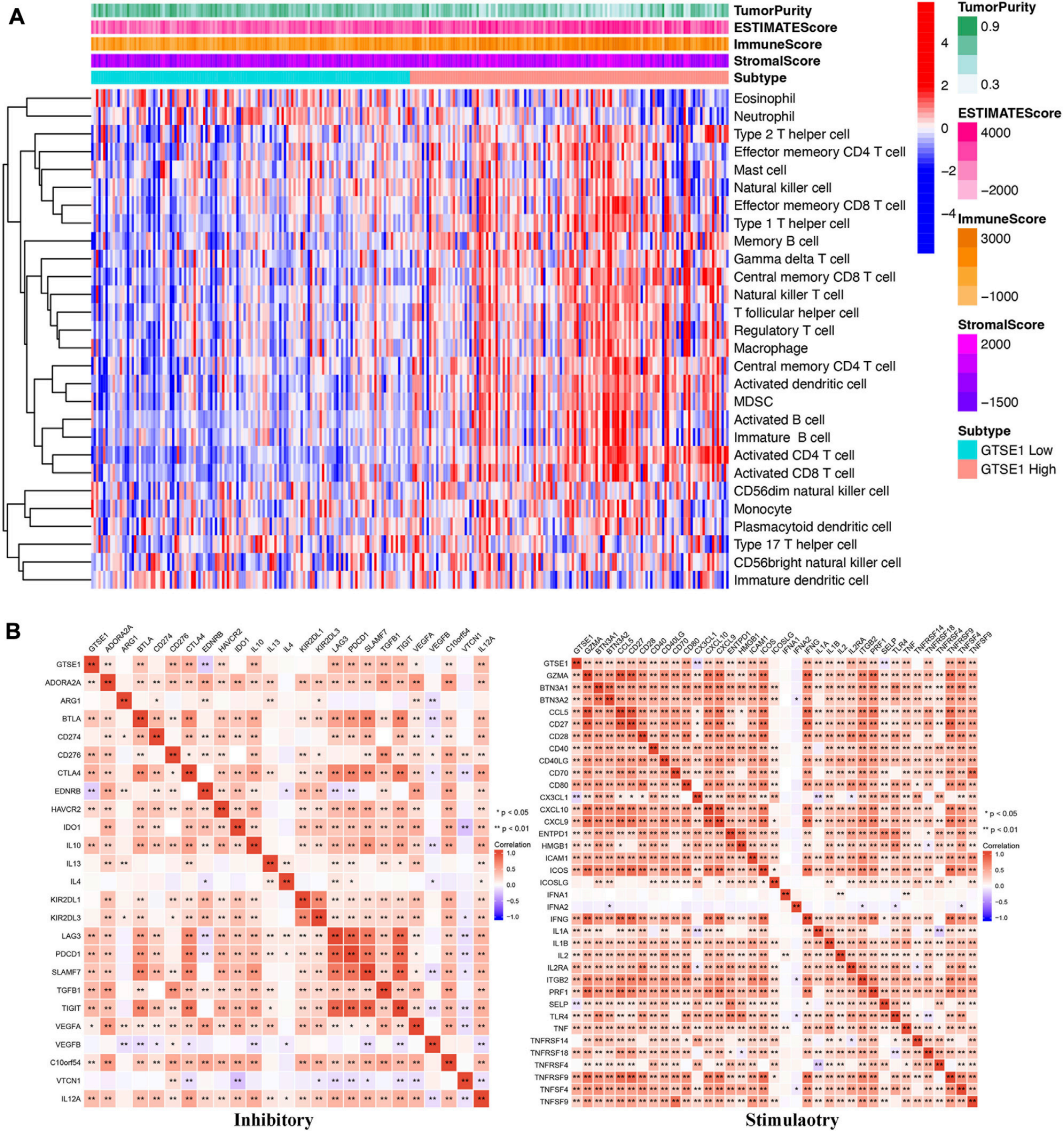
Association between GTSE1 expression and immune infiltration and immune checkpoints in ccRCC. **(A)** The correlation between the immune infiltration level and GTSE1 expression in ccRCC patients was evaluated and analyzed based on the combination of Estimate analysis and ssGSEA. **(B)** Heatmaps of the correlation between GTSE1 expression and immune checkpoints (immunoinhibitors, immunostimulators) based on the TCGA-KIRC dataset. *p < 0.05, **p < 0.01.

In recent years, the tumor microenvironment (TME) has gained increasing attention for its critical role in regulating malignant tumor progression, affecting patient prognosis, and regulating immunotherapy (Wu and Dai, 2017; Wang et al., 2018). The combination of Estimate analysis and ssGSEA was used to evaluate the correlation between the immune infiltration and GTSE1 expression (Figure 8A). The tumor purity, estimate score, immune score, stromal score, and various immune cell infiltration levels were evaluated in the high- and low-GTSE1-expression groups (cutoff by the median expression level of GTSE1). Based on the analysis results of the heatmap and the TIMER database, we concluded that GTSE1 was positively correlated with EstimateScore, ImmuneScore, StromalScore, and infiltration of multiple immune cells, such as B cells (r = 0.22, *p* = 1.9e−06), CD8^+^ T cells (r = 0.165, *p* = 5.43e−04), CD4^+^ T cells (r = 0.251, *p* = 4.89e−08), macrophages (r = 0.165, *p* = 4.44e−04), neutrophils (r = 0.285, *p* = 5.47e−10), and dendritic cells (r = 0.33, *p* = 4.99e−13), but negatively correlated with tumor purity (Supplementary Figure S4). Meanwhile, the “CIBERSORT” algorithm was used to estimate the relative infiltration proportion of 22 immune cell types in the GTSE1 high- and low-expression groups in ccRCC (cutoff by the median expression of GTSE1) based on the TCGA-KIRC cohort. The results suggested that patients with high GTSE1 expression had higher immune infiltration levels of T cells CD8, T cells follicular helper, T cells regulatory (Tregs), Monocytes, Macrophages M0, Macrophages M1, Macrophages M2, Dendritic cells resting, and Neutrophils. However, the patients with high GTSE1 expression had lower immune infiltration levels in NK cells resting, Dendritic cells activated, and Mast cells resting (Supplementary Figure S5). In brief, these results revealed that GTSE1 was positively correlated with immune cell infiltration and tumor microenvironment characteristics, especially macrophages.

### Correlation between GTSE1 expression and immune marker expression and immune checkpoints

To further clarify the association between GTSE1 expression and immune cell infiltration levels in ccRCC, the immune marker sets of various immune cells were analyzed using the TIMER and GEPIA databases. The correlation between GTSE1 expression and the expression level of biomarkers for specific immune cells, including CD8^+^ T cells, T cells (general), Tfh cells, Th1 cells, Th2 cells, Th17 cells, effector T cells, Tregs, T-cell exhaustion, dendritic cells, natural killer cells, monocytes, neutrophils, TAMs, M1 macrophages, M2 macrophages, and B cells, was assessed based on the TIMER database. The result suggested a significant correlation between the GTSE1 expression and CD8+T-cell markers (CD8A, CD8B), T-cell (general) markers (CD3D, CD3E, CD2), Tfh markers (BCL6, IL21), Th1 markers (TBX21, STAT4, STAT1, TNF, IFNG), Th2 markers (GATA3, IL13, STAT5A), effector T-cell markers (FGFBP2, FCGR3A), Treg markers (FOXP3, STAT5B, CCR8, TGFB1), T-cell exhaustion markers (PDCD1, CTLA4, LAG3, HAVCR2, GZMB), dendritic cell markers (HLA-DPB1, HLA-DRA, HLA-DPA1, CD1C, ITGAX), natural killer cell markers (KIR2DL4), monocyte markers (CD86, CD115), neutrophil markers (CCR7, CD11b), TAM markers (CD68, IL10), M1 macrophage (IRF5), M2 macrophage (CD163, VSIG4, MS4A4A), and B-cell markers (CD19, CD79A), and these correlations remained unchanged even after correction for tumor purity (Table 4). Tumor-associated macrophages (TAMs) are the most important component of the tumor microenvironment and can account for up to 50% of some solid neoplasms. Tumor-associated macrophages can promote the malignant progression of tumor cells through a variety of pathways and are currently the target cells of immunotargeted therapy (Vitale et al., 2019). The above results showed that GTSE1 expression was significantly correlated with macrophage-related cells (monocytes, TAMs, M1 macrophages, and M2 macrophages), especially monocytes and M2 macrophages. The specific correlation is shown in Supplementary Figure S6. Meanwhile, the GEPIA database was also used to further evaluate the correlation between GTSE1 and monocytes, TAMs, M1 macrophages, and M2 macrophages. Similar results were observed in the GEPIA web tools compared with those in TIMER (Table 5). These results suggested that GTSE1 might be involved in the regulation of macrophage polarization. The application of immune checkpoint inhibitors (ICIs) brings new hope for the treatment of patients with advanced ccRCC, especially advanced and metastatic ccRCC, which can significantly improve patient prognosis (Miao et al., 2018; McGregor et al., 2020). The expression of GTSE1 and the immune checkpoints, including the 24 immunoinhibitors and 36 immunostimulators, was extracted, and the correlation was analyzed based on the TCGA-KIRC cohort from the UCSC web database (Thorsson et al., 2018). The correlation analysis between the expression of GTSE1 and the immunoinhibitors and immunostimulators is shown in the form of heatmaps (Figure 8B). The results suggested that GTSE1 was strongly correlated with common immune checkpoints, including BTLA, CD276, CTLA4, LAG3, ITGIT, CD28, ITGB2, ICOS, PDCD1 (PD1), *etc.* From all of the results above, we can reasonably speculate that GTSE1 may serve as an indicator of the efficacy of immune checkpoint inhibitors due to its positive correlation with ICI expression. Even so, additional experimental analyses are needed to validate the vital role of GTSE1 in immunotherapy.

**TABLE 4:** Correlations between GTSE1 and gene markers of immune cells in TIMER.

Description	Gene markers	None		Purity	
		Cor.	p	Cor.	p
CD8+T cell	CD8A	0.34992	***	0.35248	***
	CD8B	0.31529	***	0.31888	***
T cell (general)	CD3D	0.36113	***	0.35647	***
	CD3E	0.37063	***	0.36518	***
	CD2	0.38451	***	0.37759	***
Tfh	BCL6	0.11528	**	0.10862	*
	IL21	0.20128	***	0.19370	***
Th1	TBX21	0.20800	***	0.19546	***
	STAT4	0.33749	***	0.32898	***
	STAT1	0.32971	***	0.33517	***
	TNF	0.26819	***	0.27554	***
	IFNG	0.40222	***	0.40646	***
Th2	STAT6	-0.00671	0.87713	0.01124	0.80977
	GATA3	0.12592	**	0.12597	**
	IL13	0.14219	***	0.12677	**
	STAT5A	0.26398	***	0.23410	***
Th17	STAT3	0.06623	0.12671	0.04464	0.33887
	IL17A	0.02166	0.61778	-0.00451	0.92306
Effector T-cell	CX3CR1	0.02676	0.53755	0.02205	0.63679
	FGFBP2	-0.20063	***	-0.19788	***
	FCGR3A	0.32147	***	0.32004	***
Treg	FOXP3	0.45770	***	0.45741	***
	STAT5B	-0.13954	**	-0.14747	***
	CCR8	0.36566	***	0.36996	***
	TGFB1	0.24327	***	0.19679	***
T cell exhaustion	PDCD1	0.40949	***	0.41392	***
	CTLA4	0.38155	***	0.36951	***
	LAG3	0.46010	***	0.44692	***
	HAVCR2	0.12951	**	0.13178	***
	GZMB	0.22821	***	0.21722	***
Dendritic cell	HLA-DPB1	0.21582	***	0.21881	***
	HLA-DQB1	0.09303	*	0.07432	0.11101
	HLA-DRA	0.20555	***	0.21659	***
	HLA-DPA1	0.20840	***	0.21134	***
	CD1C	0.11546	**	0.10098	*
	NRP1	-0.04587	0.29050	-0.06650	0.15400
	ITGAX	0.37992	***	0.37217	***
Natural killer cell	KIR2DL1	-0.01606	0.71151	-0.03377	0.46944
	KIR2DL3	0.01249	0.77355	0.02015	0.66605
	KIR2DL4	0.18078	***	0.16237	***
	KIR3DL1	-0.04087	0.34628	-0.01841	0.69336
	KIR3DL2	0.03513	0.41835	0.03632	0.43659
	KIR3DL3	0.06810	0.11632	0.05419	0.24554
	KIR2DS4	-0.02992	0.49061	-0.03690	0.42927
Monocyte	CD86	0.30393	***	0.30448	***
	CD115	0.30650	***	0.29626	***
Neutrophils	CCR7	0.30414	***	0.31092	***
	CD11b	0.29245	***	0.28341	***
	CD66b	-0.00164	0.96982	0.01247	0.78946
TAM	CCL2	-0.01779	0.68202	-0.05426	0.24495
	CD68	0.28870	***	0.30462	***
	IL10	0.27710	***	0.27848	***
M1 Macrophage	INOS(NOS2)	-0.00554	0.89841	-0.03098	0.50695
	IRF5	0.35396	***	0.35762	***
	COX2(PTGS2)	0.04492	0.30057	0.01424	0.76036
M2 Macrophage	CD163	0.22559	***	0.23012	***
	VSIG4	0.28625	***	0.28239	***
	MS4A4A	0.22745	***	0.22875	***
B cell	CD19	0.32425	***	0.30017	***
	CD79A	0.26782	***	0.26089	***

Cor, R value of Spearman’s correlation; None, correlation without adjustment. Purity, correlation adjusted by purity.*p < 0.05, **p < 0.01, ***p < 0.001.

**TABLE 5:** Correlation analysis between GTSE1 and relate genes and markers of monocyte, TAM and macrophages in GEPIA KIRC.

Description	Gene markers	Tumor		Normal	
		R	*P*	R	*P*
Monocyte	CD86	0.31	***	0.65	***
	CD115(CSF1R)	0.36	***	0.65	***
TAM	CCL2	−0.029	0.51	0.17	0.15
	CD68	0.30	***	0.62	***
	IL10	0.32	***	0.25	*
M1 Macrophage	INOS(NOS2)	0.10	*	0.32	**
	IRF5	0.37	***	−0.16	0.18
	COX2(PTGS2)	0.11	*	−0.11	0.38
M2 Macrophage	CD163	0.30	***	0.61	***
	VSIG4	0.33	***	0.59	***
	MS4A4A	0.28	***	0.66	***

Tumor, correlation analysis in tumor tissue of TCGA. Normal, correlation analysis in normal tissue of TCGA. *p < 0.05, **p < 0.01, ***p < 0.001.

### Effect of GTSE1 on cell proliferation in ccRCC

**FIGURE 9:**
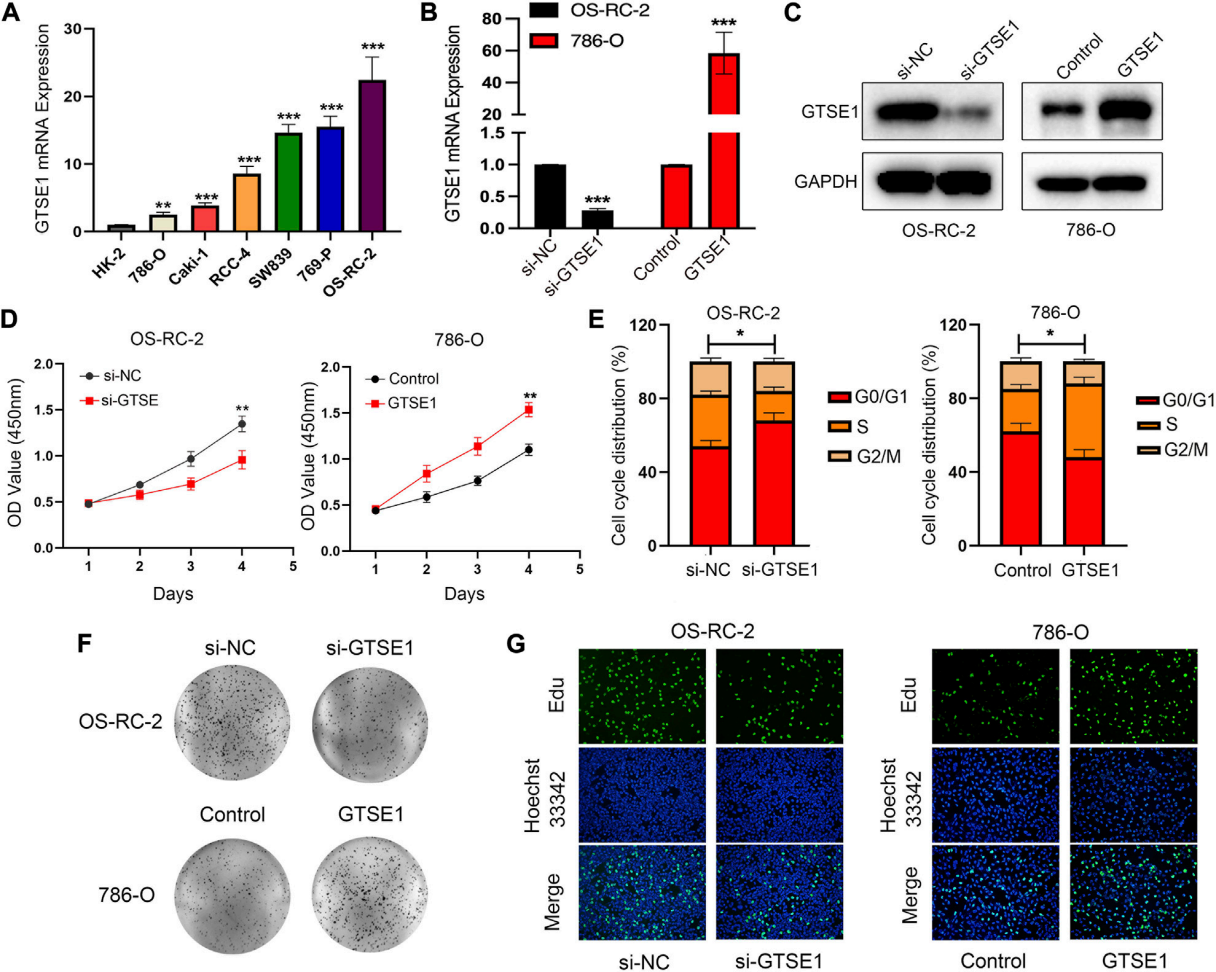
Effect of GTSE1 on cell proliferation in ccRCC. **(A)** qRT‒PCR analysis of GTSE1 mRNA expression levels in ccRCC cell lines and the human renal tubular epithelial cell line HK-2. The efficiency of GTSE1 knockdown or GTSE1 overexpression was measured by qRT‒PCR **(B)** and Western blotting **(C)** analysis in OS-RC-2 or 786-O cell lines. **(D)** Cell viability of ccRCC cells was assessed by MTT assay. **(E)** Cell cycle distribution of ccRCC cells was detected by cell flow cytometry (FCM) analysis. The results are shown as a histogram of the mean ± SD of three independent experiments. **(F)** A colony-formation assay was conducted in ccRCC cells. **(G)** DNA replication activity was assessed by an EdU-staining assay (green indicates the EdU-incorporated cells; blue indicates nuclei). GAPDH was used as an internal control. *p < 0.05, **p < 0.01, ***p < 0.001.

GTSE1 was upregulated in ccRCC tissues compared with normal tissues according to TCGA and GEO database analyses. Meanwhile, we detected GTSE1 mRNA expression in ccRCC cells and a human renal tubular epithelial cell line (HK-2) and found that compared with HK-2 cells, 786-O, Caki-1, RCC-4, SW839, 769-P, and OS-RC-2 cells had higher GTSE1 mRNA expression levels (Figure 9A). To further investigate the role of GTSE1 in regulating the malignant progression of ccRCC, gain- and loss-of-function assays were conducted by inhibiting GTSE1 expression in OS-RC-2 cells or overexpressing GTSE1 expression in 786-O cells according to GTSE1 expression in ccRCC cells. The efficiency of knockdown and overexpression of GTSE1 was measured by qRT‒PCR and Western blot assays (Figures 9B,C). The MTT assay revealed that the knockdown of GTSE1 in OS-RC-2 cells led to proliferation inhibition in a time-dependent manner. Conversely, the proliferation capacity was elevated in GTSE1-overexpressing 786-O cells (Figure 9D). A similar result was also observed in flow cytometry analysis; the results indicated that GTSE1 knockdown in OS-RC-2 cells could delay the G1/S phase transition, whereas the overexpression of GTSE1 in 786-O cells could accelerate the G1/S phase transition (Figure 9E). A colony-formation assay was also performed to confirm the clonogenic capacity of GTSE1 (Figure 9F). The DNA replication activity was detected by the EdU-staining assay, and GTSE1 suppression significantly reduced EdU staining in OS-RC-2 cells, whereas GTSE1 overexpression remarkably elevated EdU staining in 786-O cells (Figure 9G). All of these data indicated that high GTSE1 expression could promote ccRCC cell proliferation capacity.

### Effect of GTSE1 on cell migration and invasiveness in ccRCC

**FIGURE 10:**
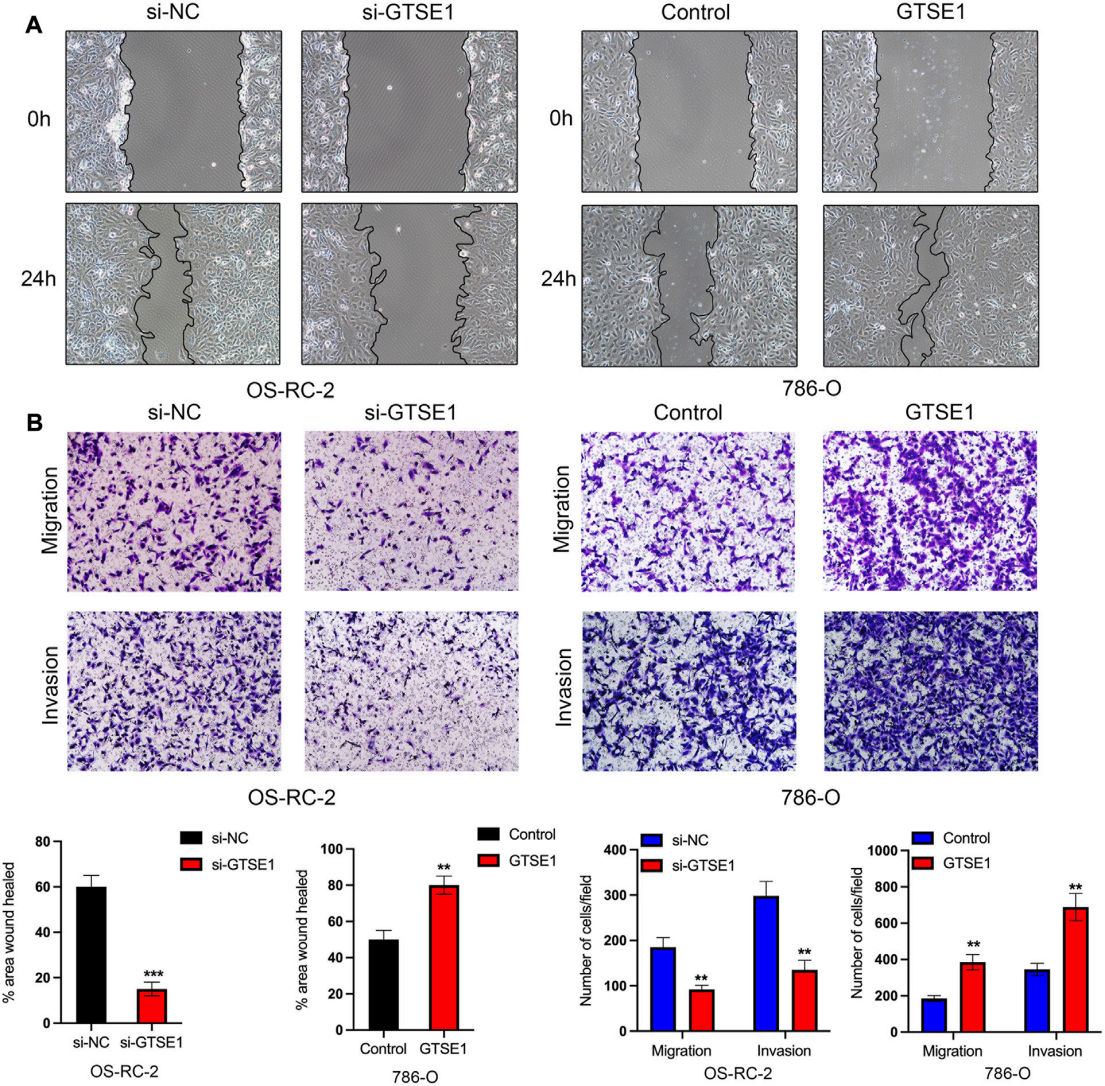
Effect of GTSE1 on cell migration and invasiveness in ccRCC **(A)** Representative images of wound healing in OS-RC-2 and 786-O cells. **(B)** Cell migration and invasion abilities were detected by transwell assays with or without Matrigel in OS-RC-2 cells with GTSE1 knockdown and in 786-O cells with GTSE1 overexpression. The quantitative analysis is shown below. **p < 0.01, ***p < 0.001.

The Gene Ontology (GO) annotation suggested that GTSE1 was probably involved in the positive regulation of cell-cell adhesion, and GSEA also found that GTSE1 could participate in the epithelial-mesenchymal transition (EMT) in ccRCC. We demonstrated that GTSE1 could promote cell proliferation in ccRCC. Furthermore, wound-healing and transwell assays were simultaneously performed to assess the potential role of GTSE1 in regulating the migration and invasion capacity of ccRCC cells. The results revealed that knocking down GTSE1 inhibited the wound-healing ability of OS-RC-2 cells and that overexpression of GTSE1 accelerated wound healing in 786-O cells (Figure 10A). Meanwhile, we also demonstrated that the GTSE1 loss resulted in an inhibition of the migration and invasion capacity in OS-RC-2 cells, whereas the GTSE1 overexpression promoted the migration and invasion ability in 786-O cells (Figure 10B). In conclusion, our results demonstrated that high GTSE1 expression could promote the migration and invasion capacity of ccRCC cells.

### Effect of GTSE1 on cisplatin sensitivity in ccRCC cells

**FIGURE 11:**
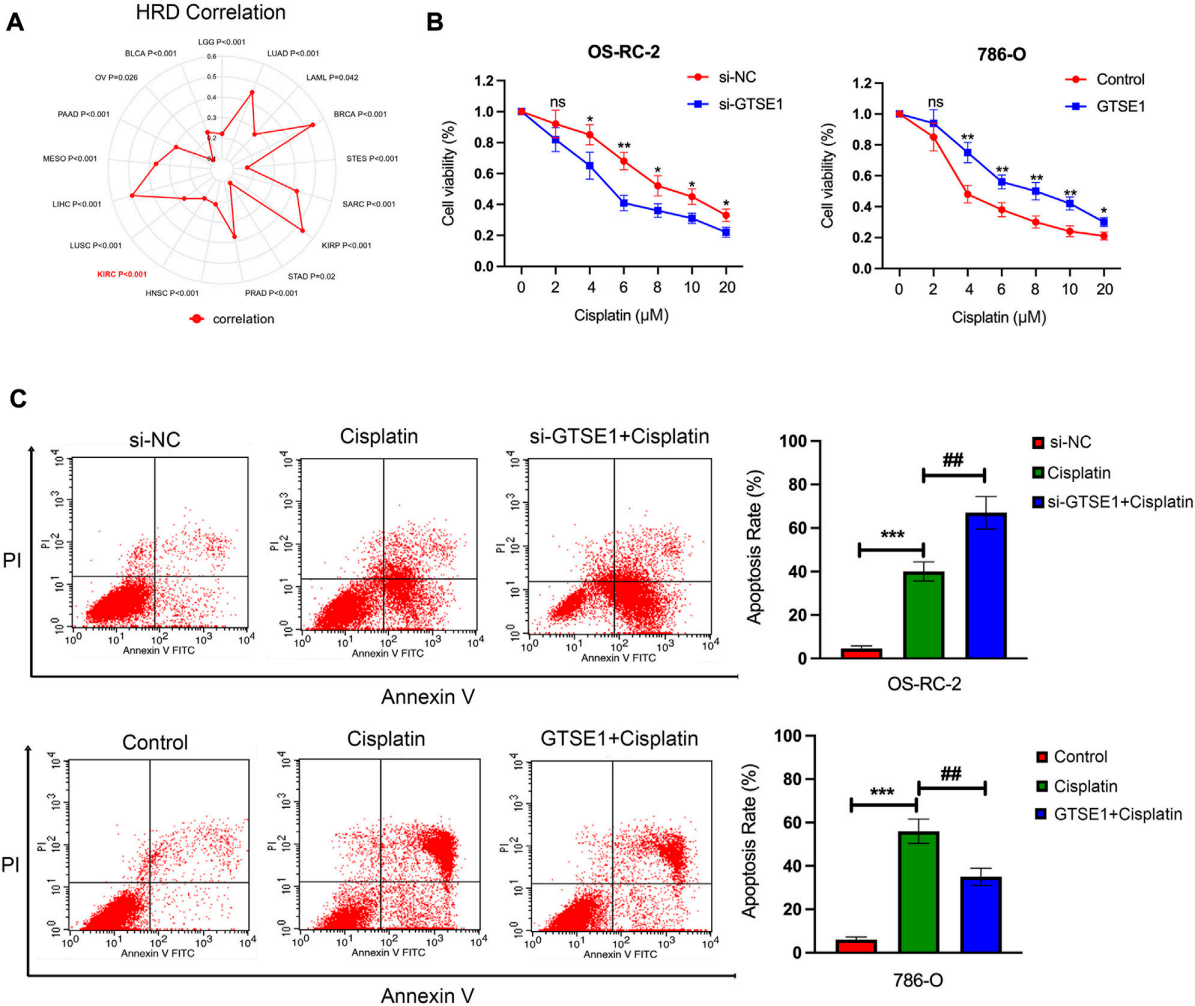
Effect of GTSE1 on cisplatin sensitivity in ccRCC cells. **(A)** The correlation between GTSE1 expression and homologous recombination deficiency (HRD) is shown in the radar map. **(B)** The chemosensitivity of ccRCC cells (OS-RC-2, 786-O) to cisplatin was measured using the MTT assay. **(C)** The cell apoptotic ratio of ccRCC cells (OS-RC-2, 786-O) was determined using the FCM assay. *p < 0.05, **p < 0.01, ***p < 0.001. ^##^ *p* < 0.01 *versus* the cisplatin treatment group.

The cellular component analysis revealed that GTSE1 had a tight relationship with microtubules, and previous research also demonstrated that GTSE1 was involved in the regulation of microtube nucleation and stability (So et al., 2019). It is well known that microtubules have a tight correlation with the chemosensitivity and chemoresistance of tumor cells (Mozzetti et al., 2005). Homologous recombination deficiency (HRD), which is a key indicator for the treatment and prognosis of various tumors, is also highly correlated with sensitivity to cisplatin and PARP inhibitors. Therefore, the correlation between HRD and GTSE1 expression across cancers was also investigated, and there was a positive correlation between HRD and GTSE1 in ccRCC (*p* < 0.001) (Figure 11A). The MTT assay also revealed that knocking down GTSE1 in OS-RC-2 cells increased the sensitivity to cisplatin, whereas the overexpression of GTSE1 in 786-O cells decreased the susceptibility to cisplatin treatment (Figure 11B). Similarly, the flow cytometry analysis also demonstrated that upregulation of GTSE1 in 786-O cells decreased, while GTSE1 ablation in OS-RC-2 cells increased the cell apoptosis ratio in ccRCC cells compared with the cisplatin treatment groups (Figure 11C). These results indicated that GTSE1 decreased the chemosensitivity of ccRCC cells to cisplatin.

## Discussion

Precision medicine has become an indispensable part of cancer treatment; thus, the discovery of biomarkers that can predict cancer prognosis and treatment efficiency is particularly urgent and required (Vargas and Harris, 2016). Due to the insensitivity of ccRCC to radiotherapy and chemotherapy, surgical resection is still the first-line therapy for clinical treatment. However, the mortality rate of postoperative patients, especially elderly patients and higher-stage patients, is still high and cannot be ignored (Falagario et al., 2021). The emergence of immune checkpoint inhibitors, such as nivolumab (anti-PD-1), has brought new hope for the treatment of ccRCC patients, but the problem of a low response rate still limits the progress of ccRCC treatment (Au et al., 2021). Therefore, the discovery of novel promising prognostic markers and therapeutic targets remains a pressing issue. Previous studies have revealed that GTSE1 could promote the malignant progression of tumors in lung cancer, colon cancer, and liver cancer (Zheng et al., 2019; Zhang F. et al., 2021; Li, 2021). However, the biological functions and underlying molecular mechanisms of GTSE1 in ccRCC progression are still poorly understood. To further understand the potential functions and regulatory network of GTSE1 in ccRCC, a series of bioinformatics analyses and functional experiments *in vitro* were performed.

In this study, bioinformatic analysis based on TCGA and GEO databases and immunohistochemistry staining on ccRCC tissue chips demonstrated that GTSE1 was particularly upregulated in ccRCC tissues and that high GTSE1 expression was significantly correlated with adverse clinicopathological factors, including advanced stage, metastasis in lymph nodes and reduced survival time in OS, DSS, and FPI, which indicated that GTSE1 might serve as an oncogene in ccRCC. Meanwhile, the high GTSE1 expression in ccRCC cells was verified by comparison with the human renal tubular epithelial cell line HK-2. To further confirm the prognostic value of GTSE1 in ccRCC, we created a nomogram to predict the OS probability in ccRCC patients based on GTSE1 expression, age, and gender, which is usually used as a predictive tool to help clinicians make clinical decisions (Cho et al., 2015). Calibration curves including 1-, 3-, and 5-year AUCs indicated that the nomogram had a high prediction accuracy, which suggested that we successfully built the GTSE1-based nomogram to guide the prognosis prediction of ccRCC patients. Despite this, the possibility of GTSE1 acting as a diagnostic or prognostic biomarker for ccRCC deserves further clinical verification.

Next, we investigated the protein-interaction network and the coexpressed genes of GTSE1, which were used for further biological functional enrichment analysis. The results suggested that the proteins that interacted with GTSE1, including CCNB2, CDK1, PLK1, and CDC20, were all associated with tumorigenic proliferation in ccRCC. Among these genes, PLK1 plays a particularly important role in cell cycle progression due to its vital role in regulating genomic stability and the DNA damage response during mitosis. Moreover, previous studies have established the causal relationship between PLK1 and tumorigenesis in ccRCC (Qian et al., 2022). Functional enrichment analysis, including GO annotation and KEGG analysis, revealed that GTSE1 was mainly located in the chromosomal region and promoted cell cycle transition and proliferation by regulating nuclear division and DNA replication. GSEA KEGG analysis also found that GTSE1 was positively correlated with cell cycle and P53 signaling pathways. All of the results above suggested that GTSE1 might serve as a regulator in the cell cycle transition and proliferation in ccRCC. Finally, gain- and loss-of-function assays were conducted to verify the oncogenic effect in promoting the proliferation of ccRCC. We found that the overexpression of GTSE1 could promote cell viability, colony formation, and cell cycle transition in ccRCC cells, while GTSE1 inhibition had the opposite effects. In addition, functional enrichment analysis also suggested that GTSE1 was associated with the positive regulation of cell-cell adhesion. GSEA also found that GTSE1 extremely participated in the epithelial-mesenchymal transition (EMT), which provided the driving force for tumor metastasis (Pastushenko and Blanpain, 2019). Meanwhile, the functional assay *in vitro* also demonstrated that the knockdown of GTSE1 suppressed, while the upregulation of GTSE1 improved, the migration and invasion capacity in ccRCC cells. All of the results above demonstrated that GTSE1 could promote the invasiveness and metastasis of ccRCC cells *in vitro*.

Considering the tight relationship between GTSE1 and microtubules and the causal association between microtubules and chemoresistance, the correlation between GTSE1 and HRD (homologous recombination deficiency) and the effect of GTSE1 on chemosensitivity to cisplatin in ccRCC were explored and demonstrated based on the TCGA-KIRC cohort and biological functional experiments. HRD is a key indicator of the treatment and prognosis of a variety of tumors. Clinical studies have confirmed that HRD status is highly correlated with sensitivity to cisplatin and PARP inhibitors (Hoppe et al., 2018; Mayer et al., 2020). The results demonstrated that GTSE1 was positively correlated with HRD in ccRCC and that the overexpression of GTSE1 could increase cell viability and decrease the apoptosis rate in ccRCC cells treated with cisplatin, while the knockdown of GTSE1 could decrease cell viability and increase the apoptosis rate in ccRCC cells treated with cisplatin. These results demonstrated that the upregulation of GTSE1 could reduce the chemosensitivity to cisplatin in ccRCC cells and ultimately contribute to chemoresistance to cisplatin in ccRCC.

ccRCC has stood out collectively of the foremost immune-infiltrated tumors, and clinically anti-PD-1/PD-L1 antibody has been permitted within the front-line setting of advanced or metastatic ccRCC (Motzer et al., 2015). Although the effect of anti-PD1 antibodies has been demonstrated, a significant proportion of patients are still non-reactive to such treatments. A recent study revealed that the state of T-cell activation in the tumor microenvironment is the prognostic determinant of ccRCC (Adotevi et al., 2010). The GO annotation and KEGG analysis all suggested that GTSE1 was significantly correlated with T-cell activation, the innate immune response, and the T-cell receptor signaling pathway. Moreover, we further explored the correlation between GTSE1 and immune infiltration in four aspects (tumor microenvironment, immune cell infiltration, immune cell markers, and immune checkpoints). In terms of the tumor microenvironment, GTSE1 was positively correlated with the StromalScore, ImmuneScore, and EstimateScore but negatively correlated with tumor purity. Regarding immune cell infiltration and immune markers, our results demonstrated that GTSE1 was not only positively correlated with immune cell infiltration, including B cells, CD8^+^ T cells, CD4^+^ T cells, macrophages, neutrophils, and dendritic cells but also positively correlated with the majority of immune cell markers. From the results of CIBERSORT, we found that GTSE1 was also positively correlated with T regulatory cells (Tregs) and macrophages (monocytes, M0 macrophages, TAMs, M1 macrophages, and M2 macrophages), in which Tregs are known as the main manipulator creating an immunosuppressive TME by suppressing the function of Th1 cells, and the higher level of Treg infiltration was related to adverse clinical-pathological factors and poor prognosis in ccRCC (Kiniwa et al., 2007; Sakaguchi et al., 2020). Meanwhile, the results in TIMER also revealed a strong positive correlation between GTSE1 and immune cell infiltration and immune marker expression of monocytes and M2 macrophages. Additionally, extensive TAMs, especially M2 macrophage infiltration, have been shown to be positively correlated with cancer progression and poor prognosis in multiple human cancers. This finding suggested that GTSE1 might play a vital role in regulating TAM polarization, which is considered one of the main regulators in the process of immune responses and is known to contribute to tumor metastasis (Noy and Pollard, 2014; Zhao et al., 2020). Coexpression analysis of GTSE1 and certain novel immune checkpoint genes indicated that high GTSE1 expression was associated with ICIs, such as PDCD1 (PD1), LAG3, and CTLA4, and might serve as an indicator for ICI therapeutic efficiency. Regardless, additional clinical trials are needed to clarify the question of whether GTSE1 could guide ICIs for further clinical application.

## Conclusion

This study identified the overexpression of GTSE1 in ccRCC, which was positively correlated with adverse clinical-pathological factors and poor prognosis. High GTSE1 expression was closely related to immune cell infiltration and gene expression of ICIs. Meanwhile, GTSE1 could also create an immunosuppressive TME by promoting the immune cell infiltration of Tregs and M2 macrophage polarization. Finally, the biological functional assay demonstrated that GTSE1 could promote the malignant progression of ccRCC by promoting proliferation, migration, invasion capacity, and cisplatin resistance in ccRCC cells. Taken together, GTSE1 could promote tumor progression and serve as a potential biomarker and prognostic predictor in ccRCC.

## Data Availability

The original contributions presented in the study are included in the article/Supplementary Material, further inquiries can be directed to the corresponding author.
