# Supplementary material for: High GTSE1 expression promotes cell proliferation, metastasis and cisplatin resistance in ccRCC and is associated with immune infiltrates and poor prognosis
Source: Front Genet. 2023 Mar 14;14:996362. doi: 10.3389/fgene.2023.996362 (PMC10043236; doi:10.3389/fgene.2023.996362)
Supplement: Supplementary file 7 [file DataSheet1.PDF]

Table 1. The correlation between GTSE1 expression and different clinical characteristics

Variables	Subgroup	No.	Comparisons	Statistical significance (*P*)
Sample	Normal	72	Normal vs. primary tumor	1.62E-12
	Primary tumor	533		
Patient's age	Normal	72		
	21-40 years	26	Normal-vs-Age(21-40Yrs)	2.75E-04
	41-60 years	238	Normal-vs-Age(41-60Yrs)	2.34E-14
	61-80 years	246	Normal-vs-Age(61-80Yrs)	1.62E-12
	81-100 years	23	Normal-vs-Age(81-100Yrs)	1.48E-03
Patient's gender	Normal	72		
	Male	345	Normal-vs-Male	1.11E-16
	Female	188	Normal-vs-Female	1.11E-16
KIRC subtypes	Normal	72	Normal-vs-ccA subtype	1.62E-12
	ccA	205	Normal-vs-ccB subtype	1.63E-12
	ccB	175	ccA subtype-vs-ccB subtype	2.39E-06
Nodal metastasis	Normal	72	Normal-vs-N0	9.99E-16
	N0	240	Normal-vs-N1	4.14E-05
	N1	16	N0-vs-N1	6.16E-04
Cancer stage	Normal	72		
	Stage 1	267	Normal-vs-Stage1	1.62E-12
	Stage 2	57	Normal-vs-Stage2	5.59E-07
	Stage 3	123	Normal-vs-Stage3	8.09E-12
	Stage 4	84	Normal-vs-Stage4	9.93E-09
Tumor grade	Normal	72		
	Grade 1	14	Normal-vs-Grade 1	4.19E-04
	Grade 2	229	Normal-vs-Grade 2	<1E-12
	Grade 3	206	Normal-vs-Grade 3	<1E-12
	Grade 4	76	Normal-vs-Grade 4	6.01E-08

Table 2. Relationship between GTSE1 expression and clinicopathological features in patients with ccRCC.

Characteristic	Low expression of GTSE1	High expression of GTSE1	*P*	method
Age, n (%)			0.245	Chisq.test
<=60	127 (23.6%)	142 (26.3%)		
>60	142 (26.3%)	128 (23.7%)		
Race, n (%)			0.420	Fisher.test
Asian	3 (0.6%)	5 (0.9%)		
Black or African American	33 (6.2%)	24 (4.5%)		
White	231 (43.4%)	236 (44.4%)		
Gender, n (%)			0.116	Chisq.test
Female	102 (18.9%)	84 (15.6%)		
Male	167 (31%)	186 (34.5%)		
T stage, n (%)			**< 0.001**	Chisq.test
T1	155 (28.8%)	123 (22.8%)		
T2	42 (7.8%)	29 (5.4%)		
T3	71 (13.2%)	108 (20%)		
T4	1 (0.2%)	10 (1.9%)		
N stage, n (%)			**0.002**	Chisq.test
N0	121 (47.1%)	120 (46.7%)		
N1	1 (0.4%)	15 (5.8%)		
M stage, n (%)			**< 0.001**	Chisq.test
M0	227 (44.9%)	201 (39.7%)		
M1	23 (4.5%)	55 (10.9%)		
Pathologic stage, n (%)			**< 0.001**	Chisq.test
Stage I	151 (28.2%)	121 (22.6%)		
Stage II	36 (6.7%)	23 (4.3%)		
Stage III	57 (10.6%)	66 (12.3%)		
Stage IV	25 (4.7%)	57 (10.6%)		
Histologic grade, n (%)			**< 0.001**	Chisq.test
G1	9 (1.7%)	5 (0.9%)		
G2	136 (25.6%)	99 (18.6%)		
G3	94 (17.7%)	113 (21.3%)		
G4	24 (4.5%)	51 (9.6%)		

Table 3. The enrichment of GSEA gene sets at both the NOM P-value <0.05 and FDR q-value <0.25.

Enrichment in phenotype: High	ES	NES	NOM P-value	FDR q-value
HALLMARK_G2M_CHECKPOINT	0.74	2.20	0.00	0.00
HALLMARK_E2F_TARGETS	0.69	2.05	0.00	0.00
HALLMARK_MITOTIC_SPINDLE	0.57	1.69	0.00	0.00
HALLMARK_IL6_JAK_STAT3_SIGNALING	0.58	1.65	0.00	0.00
HALLMARK_INFLAMMATORY_RESPONSE	0.56	1.64	0.00	0.00
HALLMARK_TNFA_SIGNALING_VIA_NFKB	0.44	1.29	0.01	0.12
HALLMARK_IL2_STAT5_SIGNALING	0.47	1.38	0.00	0.05
HALLMARK_EPITHELIAL_MESENCHYMAL_TRANSITION	0.52	1.54	0.00	0.01
Enrichment in phenotype: Low	ES	NES	NOM P-value	FDR q-value
HALLMARK_OXIDATIVE_PHOSPHORYLATION	-0.31	-1.48	0.00	0.02
HALLMARK_FATTY_ACID_METABOLISM	-0.32	-1.68	0.00	0.01
HALLMARK_PROTEIN_SECRETION	-0.29	-1.34	0.00	0.05
HALLMARK_ADIPOGENESIS	-0.28	-1.22	0.00	0.12

ES, enrichment score; FDR, false discovery rate; ES, Enrichment Score; NES, normalized enrichment score; NOM, normalized

Table 4. Correlations between GTSE1 and gene markers of immune cells in TIMER.

		None		Purity	
Description	Gene markers	Cor.	p	Cor.	p
CD8+T cell	CD8A	0.34992	***	0.35248	***
	CD8B	0.31529	***	0.31888	***
T cell (general)	CD3D	0.36113	***	0.35647	***
	CD3E	0.37063	***	0.36518	***
	CD2	0.38451	***	0.37759	***
Tfh	BCL6	0.11528	**	0.10862	*
	IL21	0.20128	***	0.19370	***
Th1	TBX21	0.20800	***	0.19546	***
	STAT4	0.33749	***	0.32898	***
	STAT1	0.32971	***	0.33517	***
	TNF	0.26819	***	0.27554	***
	IFNG	0.40222	***	0.40646	***
Th2	STAT6	-0.00671	0.87713	0.01124	0.80977
	GATA3	0.12592	**	0.12597	**
	IL13	0.14219	***	0.12677	**
	STAT5A	0.26398	***	0.23410	***
Th17	STAT3	0.06623	0.12671	0.04464	0.33887
	IL17A	0.02166	0.61778	-0.00451	0.92306
Effector T-cell	CX3CR1	0.02676	0.53755	0.02205	0.63679
	FGFBP2	-0.20063	***	-0.19788	***
	FCGR3A	0.32147	***	0.32004	***
Treg	FOXP3	0.45770	***	0.45741	***
	STAT5B	-0.13954	**	-0.14747	***
	CCR8	0.36566	***	0.36996	***
	TGFB1	0.24327	***	0.19679	***
T cell exhaustion	PDCD1	0.40949	***	0.41392	***
	CTLA4	0.38155	***	0.36951	***
	LAG3	0.46010	***	0.44692	***
	HAVCR2	0.12951	**	0.13178	***
	GZMB	0.22821	***	0.21722	***
Dendritic cell	HLA-DPB1	0.21582	***	0.21881	***
	HLA-DQB1	0.09303	*	0.07432	0.11101
	HLA-DRA	0.20555	***	0.21659	***
	HLA-DPA1	0.20840	***	0.21134	***
	CD1C	0.11546	**	0.10098	*
	NRP1	-0.04587	0.29050	-0.06650	0.15400
	ITGAX	0.37992	***	0.37217	***
Natural killer cell	KIR2DL1	-0.01606	0.71151	-0.03377	0.46944
	KIR2DL3	0.01249	0.77355	0.02015	0.66605
	KIR2DL4	0.18078	***	0.16237	***
	KIR3DL1	-0.04087	0.34628	-0.01841	0.69336
	KIR3DL2	0.03513	0.41835	0.03632	0.43659
	KIR3DL3	0.06810	0.11632	0.05419	0.24554
	KIR2DS4	-0.02992	0.49061	-0.03690	0.42927
Monocyte	CD86	0.30393	***	0.30448	***
	CD115	0.30650	***	0.29626	***
Neutrophils	CCR7	0.30414	***	0.31092	***
	CD11b	0.29245	***	0.28341	***
	CD66b	-0.00164	0.96982	0.01247	0.78946
TAM	CCL2	-0.01779	0.68202	-0.05426	0.24495
	CD68	0.28870	***	0.30462	***
	IL10	0.27710	***	0.27848	***
M1 Macrophage	INOS(NOS2)	-0.00554	0.89841	-0.03098	0.50695
	IRF5	0.35396	***	0.35762	***
	COX2(PTGS2)	0.04492	0.30057	0.01424	0.76036
M2 Macrophage	CD163	0.22559	***	0.23012	***
	VSIG4	0.28625	***	0.28239	***
	MS4A4A	0.22745	***	0.22875	***
B cell	CD19	0.32425	***	0.30017	***
	CD79A	0.26782	***	0.26089	***

Cor, R value of Spearman’s correlation; None, correlation without adjustment. Purity, correlation adjusted by purity.*$p < 0.05$, **$p < 0.01$, ***$p < 0.001$.

Table 5. Correlation analysis between GTSE1 and relate genes and markers of monocyte, TAM and macrophages in GEPIA

		KIRC			
		Tumor		Normal	
Description	Gene markers	R	*P*	R	*P*
Monocyte	CD86	0.31	***	0.65	***
	CD115(CSF1R)	0.36	***	0.65	***
TAM	CCL2	−0.029	0.51	0.17	0.15
	CD68	0.30	***	0.62	***
	IL10	0.32	***	0.25	*
M1 Macrophage	INOS(NOS2)	0.10	*	0.32	**
	IRF5	0.37	***	−0.16	0.18
	COX2(PTGS2)	0.11	*	−0.11	0.38
M2 Macrophage	CD163	0.30	***	0.61	***
	VSIG4	0.33	***	0.59	***
	MS4A4A	0.28	***	0.66	***

Tumor, correlation analysis in tumor tissue of TCGA. Normal, correlation analysis in normal tissue of TCGA. *p < 0.05, **p < 0.01, ***p < 0.001.
