# Supplementary material for: High GTSE1 expression promotes cell proliferation, metastasis and cisplatin resistance in ccRCC and is associated with immune infiltrates and poor prognosis
Source: Front Genet. 2023 Mar 14;14:996362. doi: 10.3389/fgene.2023.996362 (PMC10043236; doi:10.3389/fgene.2023.996362)
Supplement: Supplementary file 1 [file DataSheet2.PDF]

Dear Editor and Reviewers of ***Frontiers in Genetics***

October 21th, 2022

Dear editors and reviewers,

Thanks for your attention and comments on our manuscript entitled "**High GTSE1 expression promotes cell proliferation, metastasis and cisplatin resistance in ccRCC and is associated with immune infiltrates and poor prognosis**". Those comments are constructive and helpful for our revising. We have studied the comments carefully and have made corrections which we hope meet with approval. Revised portions are marked in red in the revised manuscript. The main corrections in the paper and the point-to-point responses to the reviewer's comments are listed as folllowing:

Reviewer #1:

We are all obliged to the reviewer for the valuable suggestions and comments. As can be seen from the comments, the reviewer must be an experienced researcher and have abundant knowledge reserve. We have revised the manuscript carefully according to the reviewer's comments and the revisions were highlighted with red marks. According to the reviewer's comments, changes were made in the revised manuscript. The journal formatting guidelines were followed when we submitted the revised manuscript. We then made detailed responses to the comments in the space provided.

Q1

Signaling pathways potentially involved in GTSE1 based on the KEGG analysis were as follows: cell cycle, cytokine-cytokine receptor interaction, oocyte meiosis, P53 signaling pathway, DNA replication, human T-cell leukemia virus 1 infection, Th17 cell differentiation, T cell receptor signaling pathway, primary immunodeficiency, base excision repair. In our research, we had demonstrated that GTSE1 could regulate the cell cycle by increasing cell viability and accelerating the cell cycle transition from G1 to S phase. The P53 signaling pathway greatly affected the cell cycle and tumor progression. Meanwhile, the correlation between GTSE1 and p53 signaling pathway in breast cancer progression has been demonstrated and published[1]. We also demonstrated that GTSE1 could regulate DNA replication by increasing the EdU staining signals. The main goal of chemotherapeutic drugs is to induce massive cell death in tumors. Cisplatin is an antitumor drug widely used to treat several types of cancer. The primary biological target of cisplatin is genomic DNA, and it causes a plethora of DNA lesions that block transcription and replication. These cisplatin-induced DNA lesions strongly induce

cell death if they are not properly repaired or processed[2, 3]. The base excision repair was used to counteract cisplatin-induced DNA damage. In our research, we also verified the potential effect of GTSE1 on cisplatin resistance in ccRCC. The relationship between the GTSE1 and immune cell Infiltration was poorly understood. We specified the correlation between the GTSE1 and immune-related signaling pathways such as Th17 cell differentiation, T cell receptor signaling pathway, primary immunodeficiency based on the Estimate method based on the ssGSEA and CIBERSORT method based on the LM22 analysis. Therefore, we have not verified the relationship between GTSE1 and a specific signaling pathway using molecular biological methods such as western blot. But our results also validate these correlations in another way.

1. Lin F, Xie YJ, Zhang XK, Huang TJ, Xu HF, Mei Y, Liang H, Hu H, Lin ST, Luo FF *et al*: GTSE1 is involved in breast cancer progression in p53 mutation-dependent manner. *J Exp Clin Cancer Res* 2019, 38(1):152.
2. Caiola E, Salles D, Frapolli R, Lupi M, Rotella G, Ronchi A, Garassino MC, Mattschas N, Colavecchio S, Broggini M *et al*: Base excision repair-mediated resistance to cisplatin in KRAS(G12C) mutant NSCLC cells. *Oncotarget* 2015, 6(30):30072-30087.
3. Rocha CRR, Silva MM, Quinet A, Cabral-Neto JB, Menck CFM: DNA repair pathways and cisplatin resistance: an intimate relationship. *Clinics (Sao Paulo)* 2018, 73(suppl 1):e478s.

Q2

Thank you for your suggestion on the number of figures in our article. Generally speaking, the number of pictures in the paper should not be more than 8 figures. There are the reasons my figure was more than eight pictures.

Reason 1: My study comprehensively introduced the differential expression of GTSE1 in ccRCC, its relationship with clinicopathological features, prognostic value, potential signaling pathways, and biological functions, and its correlation with the immune microenvironment and immune cell infiltration, and verified its biological function by molecular biology. This is the comprehensive study of GTSE1 in renal clear cell carcinoma. The richness and diversity of the content make the number of pictures in the article more than 8. Meanwhile, some relative data was made into tables and supplementary materials to minimize the number of pictures as much as possible.

Reason 2: The journal formatting guidelines were followed and recently similar articles published in all Frontiers journals were referenced. There are a lot of papers whose pictures are more than eight, such as[4-8] :

4. Zhang G, Li B, Lin Y: Evaluation of ITGA3 as a Biomarker of Progression and Recurrence in Papillary Thyroid Carcinoma. *Front Oncol* 2021, 11:614955.
5. Huang Y, Huang S, Ma L, Wang Y, Wang X, Xiao L, Qin W, Li L, Yuan X: Exploring the Prognostic Value, Immune Implication and Biological Function of H2AFY Gene in Hepatocellular Carcinoma. *Front Immunol* 2021, 12:723293.
6. Song Y, Jin D, Ou N, Luo Z, Chen G, Chen J, Yang Y, Liu X: Gene Expression Profiles Identified Novel Urine Biomarkers for Diagnosis and Prognosis of High-Grade Bladder Urothelial Carcinoma. *Front Oncol* 2020, 10:394.

7. Lin T, Zhang Y, Lin Z, Peng L: Roles of HMGBs in Prognosis and Immunotherapy: A Pan-Cancer Analysis. *Front Genet* 2021, 12:764245.
8. Deng M, Xiong C, He ZK, Bin Q, Song JZ, Li W, Qin J: MCTS1 as a Novel Prognostic Biomarker and Its Correlation With Immune Infiltrates in Breast Cancer. *Front Genet* 2022, 13:825901.

After repeated discussion by our research team, we believe that the number of graphics displayed in this paper is the most reasonable and optimal, although it exceeds the average number of pictures.

Q3

The sentences have been carefully revised and polished by language experts. The spelling, grammar, sentence structure, and terminology were performed through detailed proofreading. All the changes made were marked in red in the revised manuscript.

Reviewer #2:

We are all obliged to the reviewer for the valuable suggestions and comments. As can be seen from the comments, the reviewer must be an experienced researcher and have abundant knowledge reserve. We have revised the manuscript carefully according to the reviewer's comments and the revisions were highlighted with red marks. The journal formatting guidelines were followed when we submitted the revised manuscript. We then made detailed responses to the comments in the space provided.

Q1

In our research, we have demonstrated that GTSE1 could promote the cell proliferation of ccRCC by increasing cell viability and accelerating cell cycle transition. GTSE1 could also promote cell migration and invasion capacity while decreasing the chemosensitivity to cisplatin of ccRCC. In the analysis of GTSE1 expression and immune microenvironment and immune cell infiltration, the conclusion we got was mainly dependent on the bioinformatics analysis including the Estimate method based on the ssGSEA, CIBERSORT method based on the LM22 analysis, and TIMER analysis based on the specific expression of immune cell biomarkers. So far, there is no set of mature molecular biological experiments to verify the correlation between a specific gene and an immune microenvironment. Meanwhile, similar articles published in the series of "**Frontiers journals**" were referenced and similar problems also existed in these articles such as[5, 9-13]. This seems to be a common problem in the current study of the correlation between gene expression and the immune microenvironment. Of course, experimental verification is the final way to confirm the correlation, which requires the progress of our subsequent experimental technology and the improvement of analysis methods.

5. Huang Y, Huang S, Ma L, Wang Y, Wang X, Xiao L, Qin W, Li L, Yuan X: Exploring the Prognostic Value, Immune Implication and Biological Function of H2AFY Gene in Hepatocellular Carcinoma. *Front Immunol* 2021, 12:723293.
9. Zhong A, Chen T, Xing Y, Pan X, Shi M: FUCA2 Is a Prognostic Biomarker and Correlated With an Immunosuppressive Microenvironment in Pan-Cancer. *Front Immunol* 2021, 12:758648.

10. Shi J, Bao M, Wang W, Wu X, Li Y, Zhao C, Liu W: Integrated Profiling Identifies PLOD3 as a Potential Prognostic and Immunotherapy Relevant Biomarker in Colorectal Cancer. *Front Immunol* 2021, 12:722807.
11. Chen Y, Wang C, Song J, Xu R, Ruze R, Zhao Y: S100A2 Is a Prognostic Biomarker Involved in Immune Infiltration and Predict Immunotherapy Response in Pancreatic Cancer. *Front Immunol* 2021, 12:758004.
12. Zhang L, Zhang M, Wang L, Li J, Yang T, Shao Q, Liang X, Ma M, Zhang N, Jing M *et al*: Identification of CCL4 as an Immune-Related Prognostic Biomarker Associated With Tumor Proliferation and the Tumor Microenvironment in Clear Cell Renal Cell Carcinoma. *Front Oncol* 2021, 11:694664.
13. Rao J, Wu X, Zhou X, Deng R, Ma Y: TMEM205 Is an Independent Prognostic Factor and Is Associated With Immune Cell Infiltrates in Hepatocellular Carcinoma. *Front Genet* 2020, 11:575776.

My study comprehensively introduced the differential expression of GTSE1 in ccRCC, its relationship with clinicopathological features, prognostic value, potential signaling pathways, and biological functions, and its correlation with the immune microenvironment and immune cell infiltration, and verified its biological function by molecular biology. We aim to confirm the expression pattern, biological function, and regulatory role of GTSE1 in the malignant progression of renal clear cell carcinoma. Meanwhile, the correlation analysis of GTSE1 and immune microenvironment and immune cell infiltration was aimed to reveal the potential function of GTSE1 in affecting immune cell infiltration in ccRCC and to lay the foundation for whether GTSE1 could serve as a potential immunotherapeutic target in the future.

The data in this paper also indicate that GTSE1 seems to be significantly correlated with macrophage infiltration. Some preliminary experiments were planned to be conducted to clarify the potential role and molecular mechanism of GTSE1 in inducing macrophage infiltration and polarization in the ccRCC immune microenvironment and I hoped that the relevant results can be published in the journals of Frontiers. Finally, Thanks again for the reviewer's constructive comments on our article.

Q2

Thank you again for your constructive comments on this article. The long length of the paper and a large number of pictures make it look cluttered, and the amount of supplementary materials also makes it difficult to grasp the key points. But this article is still in accordance with the logic of the research. Firstly, through bioinformatics analysis and literature review, we found that GTSE1 functions as an oncogene in a variety of tumors but its biological effect in ccRCC is not well understood. We further confirmed the high expression of GTSE1 in ccRCC by immunohistochemistry of ccRCC tissue chip and TCGA and GEO data analysis. Then the correlation of GTSE1 expression and clinicopathological features and prognostic value was investigated and found that GTSE1expreesion was positively associated with adverse clinical-pathological parameters and patients with higher GTSE1expression were usually accompanied with worse outcomes. Then the relative genes that interacted with GTSE1 or co-expressed with GTSE1 were investigated and the GO and KEGG analysis based on these genes

were analyzed and found that GTSE1 not only participates in the regulation of cell proliferation, cell cycle transition, and cell-cell adhesion but also has a tight correlation with immune response. Then a series of molecular biology experiments were performed and verified the oncogenic role of GTSE1 in promoting the proliferation and metastasis of ccRCC cells. Finally, the correlations between the GTSE1 expression and immune microenvironment and immune cell infiltration were investigated by the Estimate method based on the ssGSEA, CIBERSORT method based on the LM22 analysis, and TIMER analysis based on the specific expression of immune cell biomarkers. Analysis of the relationship between gene expression homologous recombination deficiency (HRD) found that there was a positive correlation between HRD and GTSE1 in ccRCC and the GTSE1 overexpression could increase the chemotherapy-resistant of cisplatin in ccRCC cells. In conclusion, although this article is long and has many pictures, it fully elucidates the expression pattern, prognostic value, biological function, and the potential of GTSE1 as a tumor prognostic marker and immunotherapeutic target of ccRCC in the future.
